# Supplementary material for: Pyrolysis and Gasification of a Real Refuse-Derived Fuel (RDF): The Potential Use of the Products under a Circular Economy Vision
Source: Molecules. 2022 Nov 22;27(23):8114. doi: 10.3390/molecules27238114 (PMC9739972; doi:10.3390/molecules27238114)
Supplement: Supplementary file 1 [file molecules-27-08114-s001.zip › molecules-2043521-supplementary.pdf]

Pyrolysis and Gasification of a Real Refuse-Derived Fuel (RDF): The Potential Use of The Products under a Circular Economy Vision

Michela Alfè [1,2*], Valentina Gargiulo [1], Michele Porto [3], Renata Migliaccio [1], Adolfo Le Pera [4], Miriam Sellaro [4], Crescenzo Pellegrino [4], Abraham A. Abe [3], Massimo Urciuolo [1], Paolino Caputo [3], Pietro Calandra [5], Valeria Loise [3], Cesare Oliviero Rossi [3,6*], Giovanna Ruoppolo [1]

1 CNR-STEMS, National Research Council, Institute of Sciences and Technologies for Sustainable Energy and Mobility, P.le V. Tecchio 80, 80125 Napoli, Italy
2 UdR INSTM–STEMS. P.le V. Tecchio 80, 80125 Napoli, Italy;
3 Department of Chemistry and Chemical Technologies, University of Calabria, Via P. Bucci, Cubo 14/D, 87036 Rende (CS), Italy
4 Calabra Maceri e Servizi s.p.a., via M. Polo 54, 87036 Rende (CS), Italy
5 CNR-ISMN, National Research Council, Institute of Nanostructured Materials, Via Salaria km 29.300, 00015 Montelibretti (RM), Italy
6 UdR INSTM della Calabria, Via P. Bucci, Cubo 14/D, 87036 Rende (CS).
* Correspondence: michela.alfe@stems.cnr.it (M.A.); cesare.oliviero@unical.it (C.O.R.)

Table S1. Ash composition	
Element	*wt. %*
Ca	11.820
K	3.170
Na	2.675
Al	1.723
Mg	1.500
Fe	1.489
Ti	1.258
P	0.624
Ba	0.261
Mn	0.168
Cr	0.077
Cu	0.065
Sr	0.063
Sn	0.039
Sb	0.033
B	0.021
Zn	0.014
Ni	0.010
Co	0.007
Pb	0.007
V	0.003
Li	0.002
As	0.002
Mo	0.001

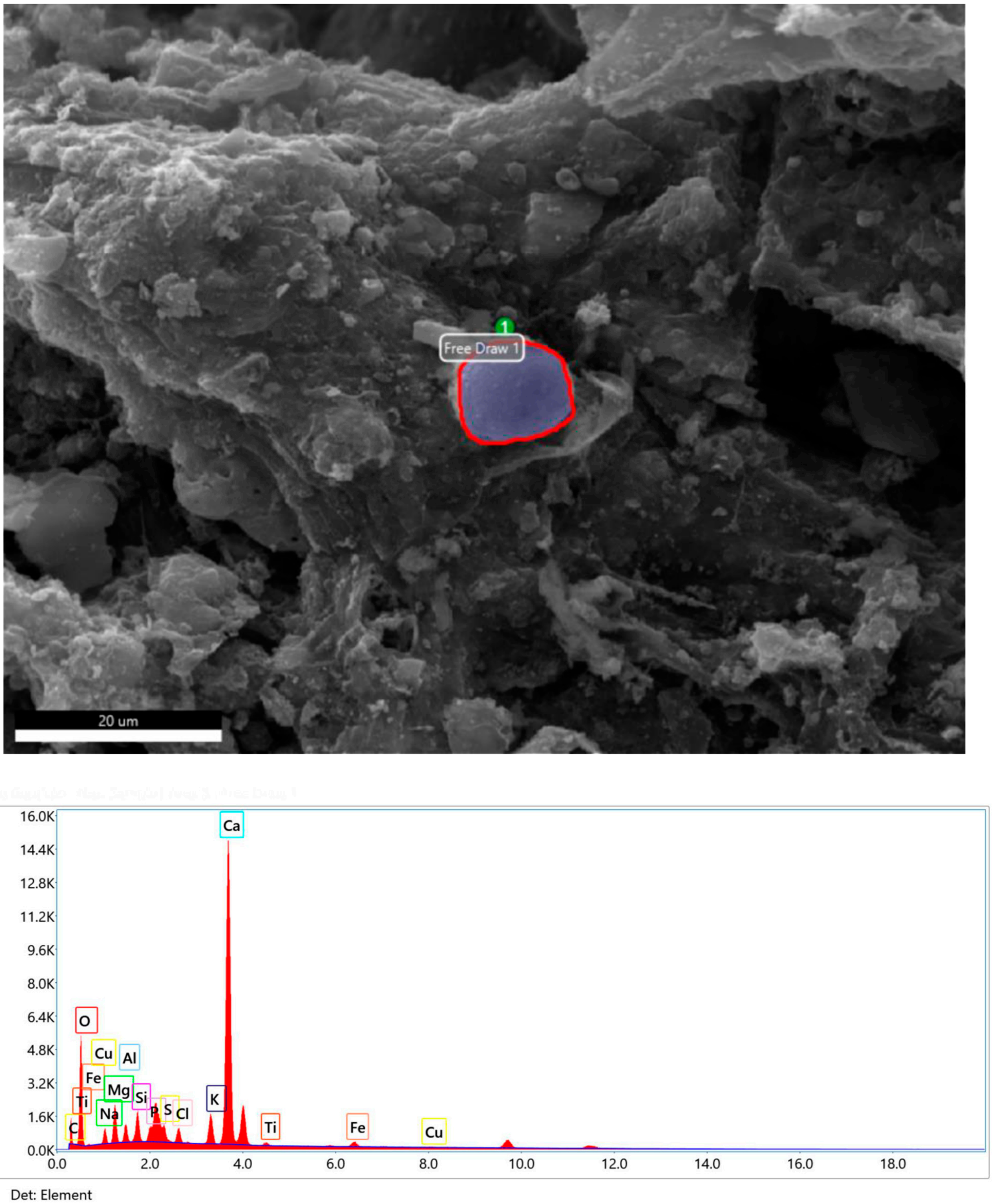

Figure S1: EDX analysis of char@750

Table S2. CS-F1@550 composition			
**RT**	**Area%**	**Library/ID**	**Quality match**
5.41	0.15	n-Hexane	45
6.98	0.57	Ethyl Acetate	86
9.31	0.2	Acetic acid	86
11.18	0.28	2-Propanone, 1-hydroxy-	50
17.73	0.14	2,4-Dimethyl-1-heptene	90
		Cyclopentane, 1,2,3-trimethyl-	53
17.74	0.13	2,4-Dimethyl-1-heptene	72
		1-Heptene, 5-methyl-	59
20.61	0.17	E-11,13-Tetradecadien-1-ol	91
		1-Nonene	80
		1-Hexene, 3-methyl-	72
20.92	0.16	Nonane	90
22.08	0.15	Bicyclo[4.2.0]octa-1,3,5-triene	94
		1,3,5,7-Cyclooctatetraene	93
22.66	0.13	2-Furanmethanol	96
26.45	0.63	1-Decene	96
		Cyclodecane	90
26.72	0.23	Decane	94
		Undecane	90
27.70	0.13	Benzaldehyde	72
29.78	0.17	Benzonitrile	91
		2-Ethynyl pyridine	90
31.48	0.23	4-Methyl-2-heptene	45
		3-Heptene, 4-methyl-	43
		Cyclohexane, 1-ethyl-2-propyl-	43
31.71	0.11	Cyclopropane, 1-butyl-2-pentyl-, cis-	58
		Cyclopropane, 1-butyl-2-pentyl-, trans-	52
		s-Tetrazine, 3,6-bis(dimethylamino)-	50
31.72	0.09	1-Heptanol, 6-methyl-	45
		3-Heptene, 4-methyl-	43
32.00	1.03	1-Undecene	91
		7-Tetradecene, (Z)-	90
		3-Tetradecene, (Z)-	90
32.24	0.49	Undecane	96
32.46	0.17	Phenol	94
33.84	0.31	Acetophenone	91
35.46	0.05	Phenol, 3-methyl-	91
		p-Cresol	90
35.47	0.04	Phenol, 2-methyl-	90
		Phenol, 3-methyl-	87
35.55	0.08	1,6-Heptadiene, 3,5-dimethyl-	49
		1,7-Nonadiene, 4,8-dimethyl-	47
		Hexanenitrile, 5-methyl-	43

36.53	0.49	1,2-Propanedione, 1-phenyl-	70
		N-Methoxy-N-methylbenzamide	64
		Benzoylformic acid	64
36.99	0.56	9-Octadecen-1-ol, (Z)-	86
		Cyclododecene	55
		1,13-Tetradecadiene	50
37.21	1.18	1-Dodecene	93
		3-Tetradecene, (Z)-	91
		5-Tetradecene, (E)-	91
37.43	0.82	Dodecane	95
37.82	0.25	Pentanedinitrile, 2-methylene-	90
		3-Hexenedinitrile	87
38.08	0.45	Cyclopropyl carbinol	42
38.28	0.23	2-Norbornanone	50
38.42	0.22	Benzoic acid, ethyl ester	81
38.79	0.25	Pentanedinitrile	72
		Cyclopropane, 1-propenyl-	72
39.22	0.38	Pentanedinitrile, 2-methyl-	83
		Hexanedinitrile	47
39.41	0.15	D-Alanine, N-allyloxycarbonyl-, decyl ester	52
		Azulene	46
39.82	0.05	Phenol, 2,6-dimethyl-	96
		Phenol, 2,5-dimethyl-	93
40.13	0.08	Ethanone, 1-(3-methylphenyl)-	72
		Ethanone, 1-(4-methylphenyl)-	70
41.396	7.13	Benzoic acid	96
41.591	0.03	Benzoic acid	94
41.921	0.27	1-Pentadecyne	92
		11-Hexadecen-1-ol, acetate, (Z)-	70
		Bicyclo[4.1.0]heptane, 2-methyl-	60
42.094	1.39	1-Tridecene	98
42.285	0.89	Tridecane	97
42.93	0.9	Cyclopentane, (2-methylbutyl)-	46
		Cyclohexane, 1,2,4-trimethyl-	43
43.33	0.29	1-Ethyl-2,2,6-trimethylcyclohexane	43
		Cyclopentane, hexyl-	42
		Cyclohexane, 1,2,4-trimethyl-	41
43.73	0.92	Cyclohexane, 1,1-dimethyl-2-propyl	46
		4-Isopropyl-1,3-cyclohexanedione	38
44.81	0.14	Benzocycloheptatriene	86
		1H-Indene, 1-ethylidene-	64
		Naphthalene, 1-methyl-	62
45.01	0.1	3-Decene, 2,2-dimethyl-, (E)-	38
		Cyclohexane, 1,1,2-trimethyl-	38
45.39	0.1	1H-Inden-1-one, 2,3-dihydro-	70
		Pyrazolo(2,3-a)pyridine, 7-methyl-	41

45.58	0.17	1-Undecene, 5-methyl-	44
		Naphthalene, 1-methyl-	35
46.05	0.21	Benzoic acid, 4-methyl-	95
46.32	0.12	1,2-Benzenedicarbonitrile	60
		1,3-Dicyanobenzene	60
46.44	0.32	Oxiranecarboxaldehyde, 3-methyl-3-(4-methyl-3-pentenyl)-	49
		7-Tetradecene	45
46.53	0.39	Z,Z-2,15-Octadecedien-1-ol acetate	94
		1,12-Tridecadiene	93
		cis-9-Tetradecen-1-ol	87
46.69	2.19	1-Tetradecene	98
		2-Tetradecene, (E)-	98
46.851	1.13	Tetradecane	98
47.16	0.17	Cyclotetradecane	56
		3-Tetradecene, (E)-	49
		Cyclopentane, nonyl-	46
47.71	0.14	1-Propanol, 2,2-dimethyl-, benzoat	76
		Ethanol, 2,2,2-trichloro-, benzoat	38
48.455	1.93	Biphenyl	95
49.99	0.09	Benzonitrile, 4-acetyl-	70
		3-Acetylbenzonitrile	70
50.22	0.29	Methanol, oxo-, benzoate	47
		2-Chloroethyl benzoate	46
		Benzoic acid, ethyl ester	46
50.65	0.85	Diphenylmethane	96
		1,1'-Biphenyl, 2-methyl-	83
50.88	0.48	9-Octadecen-1-ol, (Z)-	94
		11-Hexadecen-1-ol, (Z)-	91
51.02	2.32	1-Pentadecene	98
51.15	1.29	Pentadecane	97
		Heptadecane	91
52.01	0.08	Cycloheptane, methyl-	55
52.55	0.59	Cyclohexane, 1,2,4-trimethyl-	42
		Cyclohexane, 1,1,2-trimethyl-	41
52.98	0.3	1,1'-Biphenyl, 4-methyl-	94
		1,1'-Biphenyl, 3-methyl-	92
53.42	0.34	1,1'-Biphenyl, 3-methyl-	96
		1,1'-Biphenyl, 4-methyl-	93
53.67	0.2	2-Methyl-2-docosene	68
		9-Tricosene, (Z)-	58
		Ethanone, 1-cyclopentyl-	46
54.32	0.07	1-Pentadecene	46
		Cyclopentane, 1-butyl-2-ethyl-	35
54.99	0.57	1,9-Tetradecadiene	93
		9-Octadecen-1-ol, (E)-	93
55.11	2.31	Cetene	98

55.23	1.3	Eicosane	98
		Hexadecane	98
55.68	0.25	Bicyclo[2.2.1]heptane-2,5-dione, 1,7,7-trimethyl-	62
		3-Hexene, 2,2,5,5-tetramethyl-, (Z)-	49
		Borinic acid, diethyl-, 1-cyclododecen-1-yl ester	38
56.08	0.08	Cyclopentane, 1,1'-[3-(2-cyclopentylethyl)-1,5-pentanediyl]bis-	70
		3-Tetradecene, (Z)-	60
		Cyclohexadecane	58
56.59	0.21	4-Methyl-2,6-dihydroxyquinoline	43
		3-Hydroxy-4-phenyl-5-butyl-1,2,4-triazole	43
		2H-1-Benzopyran-2-one, 7-amino-4-methyl-	38
57.79	0.61	Fluorene	91
58.03	0.1	Cyclopentane, 1-pentyl-2-propyl-	52
		Cyclopentanone, 3-(6,6-dimethyl-5-oxo-2-heptenyl)-, (E)-	45
		Trifluoroacetoxy hexadecane	44
58.40	0.17	Isophthalic acid, ethyl 2-methoxyethyl ester	64
		Terephthalic acid, 3,5-difluorophenyl ethyl ester	64
		Isophthalic acid, 2,5-dimethylphenyl ethyl ester	59
58.67	0.43	8-Heptadecene	83
		Cyclotetradecane	74
		2- Chloropropionic acid, hexadecylester	64
58.76	0.2	Cyclopentadecane	90
		7-Tetradecene, (Z)-	86
		Z-5-Nonadecene	83
58.98	2.8	E-14-Hexadecenal	99
		3-Heptadecene, (Z)-	99
		1-Heptadecene	98
59.08	1.28	Heptadecane	98
59.315	0.22	1-Heneicosyl formate	90
		1-Eicosanol	90
		1-Nonadecene	89
59.70	0.16	Octatriacontyl pentafluoropropionate	53
		Hexatriacontyl pentafluoropropionate	53
		Tetrapentacontane, 1,54-dibromo-	53
59.79	0.09	9-Tricosene, (Z)-	98
		Z-11-Tetradecen-1-ol trifluoroacetate	97
		2,6,10,14-Tetramethyl-7-(3-methylpent-4-enylidene) pentadecane	93
59.97	1.49	Benzophenone	95
60.252	0.15	Cyclopropane carboxamide, 2-cyclopropyl-2-methyl-N-(1-cyclopropylethyl)-	38
		2-Propanone, 3-chloro-1-(1,1-dimethylethyl)-1-phenylthio-	35
60.75	0.13	9-Tricosene, (Z)-	74
		E-11(13-Methyl)tetradecen-1-ol acetate	64
60.94	0.47	Cyclohexane, 1-ethyl-2-propyl-	38
		Cyclohexane, (1-octylnonyl)-	35
61.71	0.19	1-Pentadecene	62
		3-Hexadecene, (Z)-	58

		2-Methyl-2-docosene	55
61.97	0.35	Sulfurous acid, octadecyl 2-propyl ester	38
		Nonane, 2-methyl-3-methylene-	30
		Cyclopentane, propyl-	30
62.66	4.68	E-15-Heptadecenal	99
		1-Octadecene	99
62.89	0.11	1-Cyclohexylnonene	70
		1,3-Dioxolane, 4-ethyl-5-octyl-2,2-bis(trifluoromethyl)-, trans-	53
		1,19-Eicosadiene	49
62.94	0.15	2-Methyl-2-docosene	84
		4-Methyl-dodec-3-en-1-ol	52
		Elaidic acid, isopropyl ester	49
63.15	0.37	2-Methyl-2-docosene	83
		9-Tricosene, (Z)-	64
		5-Octadecene, (E)-	45
63.48	0.37	5,8-Methano-4H-3,1-benzoxazine-2-thione, 1,2,4a-rel,5-trans,8-trans,8a-cis-hexahydro-	42
		Acetamide, 2,2,2-trifluoro-N-(3-methyl-4-nitro-5-pyrazolyl)-	42
		Propanedinitrile, (1-methylethylidene)-	38
63.79	0.39	11,13-Dimethyl-12-tetradecen-1-olacetate	64
		6,11-Dimethyl-2,6,10-dodecatrien-1-ol	45
		Cyclohexane, 1,1,3,5-tetramethyl-,trans-	38
64.28	0.8	Hexanedinitrile	64
		Pentanedinitrile, 2-methyl-	64
		p-Benzoquinone	53
64.48	0.2	[1,2,4]Triazolo[1,5-a]pyrimidin-7-ol, 6-amino-5-methyl-2-propyl-	0 49
		[1,1'-Biphenyl]-2-carbonitrile	46
		Imidazolo[1,2-a]pyrimidine-2,5(1H,3H)-dione, 3,7-dimethyl-	42
64.86	0.51	9H-Fluoren-9-one	87
65.24	0.16	1-Tricosene	90
		9-Tricosene, (Z)-	86
		7-Hexadecene, (Z)-	64
65.55	0.53	Silacyclopen-2-ene, 1,1-difluoro-	53
		2-Pentenenitrile	53
65.84	0.13	7,8-Diphenylbicyclo[4.2.1]nona-2,4,7-triene	83
		1,10b(2H)-Dihydropyrano[3,4,5-jk]fluorene	70
		Ethanone, 2-imino-1,2-diphenyl-, semicarbazone	60
65.97	0.2	2-Methyl-2-docosene	94
		9-Tricosene, (Z)-	56
		Z-5-Nonadecene	55
66.15	4.34	1-Nonadecene	99
		Z-5-Nonadecene	98
		1-Heptadecene	95
66.4	0.27	9-Tricosene, (Z)-	80
		Octadec-9-enoic acid	78
67.56	0.19	Ethanone, 1-[1,1'-biphenyl]-4-yl-	70

		Ethanone, 1-[1,1'-biphenyl]-3-yl-	55
68.30	0.3	2-Methyl-2-butenenitrile	68
		3-Butenenitrile, 2-methyl-	64
		Butanenitrile, 2-methylene-	58
68.50	0.24	Pyridine-3-carboxamide, oxime, N-(2-trifluoromethylphenyl)-	91
		2-Methyl-2-docosene	46
		Cyclohexane, 1-ethyl-2-propyl-	45
69.45	0.33	2-Methyl-2-docosene	89
		Pyridine-3-carboxamide, oxime, N-(2-trifluoromethylphenyl)-	80
		Cyclopentane, 1,1'-[3-(2-cyclopentylethyl)-1,5-pentanediyl]bis-	80
69.67	4.22	9-Tricosene, (Z)-	99
		5-Eicosene, (E)-	96
		Pentafluoropropionic acid, octadecyl ester	94
69.94	0.2	Oxacyclotetradecan-2-one, 14-methyl	70
		5-Octadecene, (E)-	48
		13-Borabicyclo[7.3.0]tridecane, 13-butoxy-, (Z)- or (E)-	45
71.43	0.27	2-Ethylacridine	38
		Benzene, 2-[(tert-butyldimethylsilyl)oxy]-1-isopropyl-4-methyl-	38
71.63	0.4	11,13-Dimethyl-12-tetradecen-1-ol acetate	64
		1-Cyclohexyl-1-(4-methylcyclohexyl)ethane	46
		6-Nitroundec-5-ene	43
72.99	3.2	Hexatriacontyl pentafluoropropionate	90
		Octatriacontyl pentafluoropropionate	90
		Octacosyl trifluoroacetate	86
73.69	0.25	Pyridine-3-carboxamide, oxime, N-(2-trifluoromethylphenyl)-	90
		Hexahydropyridine, 1-methyl-4-[4,5-dihydroxyphenyl]-	70
		1H-Indole, 5-methyl-2-phenyl-	38
73.98	4.17	Z-5-Nonadecene	94
		1-Nonadecene	94
		Trifluoroacetoxy hexadecane	93
74.30	0.3	1-Heneicosanol	91
		9-Tricosene, (Z)-	59
77.90	0.11	2,4-Cyclohexadien-1-one, 3,5-bis(1,1-dimethylethyl)-4-hydroxy-	74
		1,2-Dihydroanthra[1,2-d]thiazole-2,6,11-trione	44
		Cyclohexane, 1,1'-(2-propyl-1,3-propanediyl)bis-	43
8.42	0.31	Pyridine-3-carboxamide, oxime, N-(2-trifluoromethylphenyl)-	96
		Fumaric acid, pent-4-en-2-yl tridecyl ester	38
79.50	4	1-Docosene	97
		11-Tricosene	97
		Cycloeicosane	93
79.90	0.36	1,3-Dioxolane, 4-ethyl-5-octyl-2,2-bis(trifluoromethyl)-, cis-	50
		5-Methyl-Z-5-docosene	45
		1,3-Dioxolane, 4-ethyl-5-octyl-2,2-bis(trifluoromethyl)-, trans-	43
82.14	0.48	p-Terphenyl	55
		m-Terphenyl	42
83.78	0.51	Bicyclo[3.1.1]heptan-3-one, 6,6-dimethyl-2-(2-methylpropyl)-	46

84.17	0.71	S-Benzoyl-N-(p-methoxybenzylidene) thiohydroxylamine	58
		3-Benzoylamino-2-methyl-butyric acid, ethyl ester	52
		4-(3-Acetylphenyliminomethyl)phenyl benzoate	52
85.31	0.2	m-Terphenyl	56
		p-Terphenyl	42
86.68	3.69	1-Nonadecene	95
		1-Decanol, 2-hexyl-	91
		11-Tricosene	89
95.25	1.25	Octatriacontyl pentafluoropropionate	91
		1-Hexadecanol, 2-methyl-	90
		Tetratriacontyl pentafluoropropionate	64
95.28	0.6	Eicosane	64
		Tetratriacontyl heptafluorobutyrat	60
		Octacosyl trifluoroacetate	60
95.45	0.76	9-Tricosene, (Z)-	92
		1-Tricosene	84
96.12	3.69	1-Nonadecene	95
		Octatriacontyl pentafluoropropionate	91
		Dotriacontyl heptafluorobutyrate	91
101.75	0.17	9-Tricosene, (Z)-	90
105.02	0.48	9-Tricosene, (Z)-	91
		Fumaric acid, 2,4,6-trichlorophenyl tridecyl ester	42
108.61	1.93	1-Tricosene	95
		Tetratriacontyl pentafluoropropionate	93
		Tetratriacontyl heptafluorobutyrat	93
108.64	1.59	1-Nonadecene	96
		Octatriacontyl pentafluoropropionate	93
		Octacosyl trifluoroacetate	93
	1.54	Others	

Table S3. CS-F2@550 composition

RT	Area%	Library/ID	Qual
5.41	0.26	n-Hexane	38
6.58	0.21	Cyclopentane, methyl-	45
		1-Pentene, 2-methyl-	38
6.98	1.37	Ethyl Acetate	86
9.29	3.17	Acetic acid	90
11.18	3.61	2-Propanone, 1-hydroxy-	59
13.98	0.17	Pyridine	91
14.44	0.19	Propanoic acid	91
16.92	0.33	2-Butoxyethyl acetate	33
18.84	0.57	Propanoic acid, 2-oxo-, methyl ester	72
		Butyric acid hydrazide	39
20.62	0.5	Pent-2-ynal	72
		2-Cyclopenten-1-one	68
22.10	0.1	Styrene	93
		1,3,5,7-Cyclooctatetraene	87
22.66	2.3	2-Furanmethanol	98
23.11	0.31	2-Butanone, 3-methyl-	53
		2-Propanone, 1-(acetyloxy)-	45
		Acetic acid ethenyl ester	38
26.36	1.56	2-Cyclopenten-1-one, 2-hydroxy-	80
		1,2-Cyclopentanedione	72
		Cyclohexanone	64
26.75	0.45	Cycloheptane	38
		Nonahexacontanoic acid	38
		2-Cyclohexen-1-ol	38
27.72	0.81	Benzaldehyde	90
27.94	0.98	2(5H)-Furanone	86
		2-Butenal, 2-methyl-	43
28.43	0.35	2-Furancarboxaldehyde, 5-methyl-	87
29.08	0.35	Cyclopropane, methylmethylene-	91
		2-Cyclopenten-1-one, 3-methyl-	90
		2-Cyclopenten-1-one, 2-methyl-	87
29.79	0.84	Benzonitrile	95
29.96	0.27	4(1H)-Pyrimidinone, 6-methyl-	64
		1H-Imidazole-2-carboxaldehyde, 1-methyl-	47
		Resorcinol	46
31.49	0.17	1-Heneicosyl formate	74
		Cyclooctane, methyl-	52
		Cyclopentane, propyl-	52
32.05	1.79	2-Cyclopenten-1-one, 2-hydroxy-3-methyl-	94
		1,2-Cyclopentanedione, 3-methyl-	91
32.25	0.38	Undecane	91
		Dodecane	80

32.47	0.52	Phenol	94
		Phosphonic acid, (p-hydroxyphenyl)	90
33.84	1.11	Acetophenone	94
34.59	0.18	Benzoic acid, methyl ester	70
35.17	0.51	Phenol, 2-methoxy-	87
		Mequinol	81
35.47	0.13	Phenol, 2-methyl-	94
		Phenol, 3-methyl-	91
35.93	0.44	Propanedinitrile, dimethyl-	50
		Propanedinitrile, (1-methylpropylidene)-	43
		2-Methoxy-3-nitropyridine	22
36.54	2.13	S-Benzoyl-N-(p-nitrobenzylidene)thiohydroxylamine	53
		Benzenepropanenitrile, .beta.-oxo-	53
		Benzeneacetic acid, .alpha.-oxo-,methyl ester	53
36.94	1.46	Phenol, 3-methyl-	83
		Phenol, 2-methyl-	55
37.22	0.85	Cyclododecane	91
		1-Tetradecene	87
		Pentafluoropropionic acid, decyl ester	86
37.43	0.69	Dodecane	93
37.84	1.06	3-Hexenedinitrile	91
		Pentanedinitrile, 2-methylene-	91
38.10	2.79	Glutaraldehyde	38
		1-Isopropyldiaziridine	38
		Cyclopropyl carbinol	36
38.29	1.48	3-Hexenedinitrile	52
		3-Penten-1-yne, (E)-	43
		1-Penten-3-yne	43
38.43	0.47	Benzoic acid, ethyl ester	91
38.81	1.03	Pentanedinitrile	83
		Cyclobutanecarbonitrile	78
		2-Pentenenitrile	72
39.23	2.07	Pentanedinitrile, 2-methyl-	91
39.40	0.21	Naphthalene	60
		l-Alanine, N-allyloxycarbonyl-, butyl ester	58
40.31	0.28	Pentanedinitrile, 2-methyl-	38
		Bicyclo[3.1.0]hexan-2-one	32
41.34	8.02	Benzoic acid	96
41.65	0.13	Benzoic acid	94
41.93	0.38	Cyclododecene	43
		1,11-Dodecadiene	41
		1,2,4-Triazol-4-amine, 5-methyl-3-(3,5-dimethylpyrazol-1-yl)-	38
42.11	0.98	1-Tridecene	99
		Cyclododecane	91
42.30	1.04	Tridecane	96
42.94	0.91	Cyclohexane, 1,1-dimethyl-2-propyl	43

		1-Hexene, 2,5,5-trimethyl-	43
		Cyclohexane, 1,2,3-trimethyl-, (1.alpha.,2.beta.,3.alpha.)-	41
43.34	0.84	Fluoren-9-ol, 3,6-dimethoxy-9-(2-phenylethynyl)-	43
		Silane, (1,1-dimethylethyl)dimethyl(nonadecyloxy)-	27
		(3-Amino-6-thiophen-2-yl-thieno[2,3-b]pyridin-2-yl)-thiophen-2-yl-methanone	27
43.72	2.03	4-Octene, 2,3,6-trimethyl-	50
		Cyclohexane, 1,1,4,4-tetramethyl-	46
		1-Pentene, 3,3-dimethyl-	46
45.43	0.11	2(3H)-Furanone, dihydro-5-pentyl-	53
		2(3H)-Furanone, 5-ethyldihydro-	52
		2,2-Dimethylvaleroyl chloride	50
45.46	0.03	4,7,7-Trimethyl-5-(tetrahydropyran-2-yloxy)-bicyclo[2.2.1]heptan-2-one	50
		Tetrahydrofuran, 2-methyl-5-pentyl	50
		2H-Pyran-2-ol, tetrahydro-	47
45.69	0.46	5-Hydroxymethylfurfural	52
		4-Mercaptophenol	43
		2-Pyrazoline, 5-ethyl-1,4-dimethyl	38
46.06	0.11	Benzoic acid, 4-methyl-	70
46.08	0.12	Benzoic acid, 4-methyl-	93
46.53	0.39	1,13-Tetradecadiene	97
		1-Cyclohexylheptene	87
46.70	1.5	2-Tetradecene, (E)-	98
		1-Tetradecene	97
		Cyclotetradecane	90
46.86	0.62	Tetradecane	98
48.47	1.14	Biphenyl	81
50.24	0.55	2-Chloroethyl benzoate	93
		Benzoic acid, ethyl ester	58
50.66	0.18	Diphenylmethane	93
50.89	0.09	5-Nonadecen-1-ol	78
		Undecanenitrile, 11-bromo-	70
		(4-Methyl-pent-3-enyl)-cyclohexane	64
51.03	1.18	1-Pentadecene	97
51.17	0.42	Pentadecane	96
52.34	0.31	Ethyl 4-cyanobenzoate	81
		Benzoic acid, 4-cyano-	68
52.57	0.99	Benzamide, N-(aminocarbonyl)-	55
		Phenol, 2-methoxy-3-(2-propenyl)-	45
54.99	0.11	13-Oxabicyclo[10.1.0]tridecane	91
		1-Cyclohexylheptene	90
		E,Z-2,13-Octadecadien-1-ol	90
55.11	0.88	Cetene	96
		Z-8-Hexadecene	95
		Pentafluoropropionic acid, 4-hexadecyl ester	94
55.23	0.46	Tetracosane	97
		Hexadecane	96

56.60	0.52	Precocene I	38
		4-Methyl-2,6-dihydroxyquinoline	38
57.76	0.28	3-tert-Butyl-4-hydroxyanisole	38
		1-(m-Dimethylaminophenyl)ethanol	30
		Ethylene, 1,1-diphenyl-	30
57.82	0.5	Benzothiazol-2-amine, 6-methoxy-N-(4-methylbenzylideno)-	53
		Fluorene	38
58.40	0.25	Terephthalic acid, 3,5-difluorophenyl ethyl ester	72
		Terephthalic acid, monochloride, ethyl ester	64
		Terephthalic acid, 3-chlorophenylethyl ester	59
58.53	0.39	Diethyl Phthalate	97
58.65	0.1	3,4-Altrosan	30
		.beta.-D-Glucopyranose, 1,6-anhydro-	30
58.99	0.93	1-Heneicosyl formate	98
		3-Heptadecene, (Z)-	97
		8-Heptadecene	95
59.08	0.51	Heptadecane	96
		Eicosane	95
59.99	0.45	Benzophenone	93
		Bicyclo[3.2.1]octane-6-carboxylic acid, 2-oxo-, methyl ester, (6S)-	91
60.25	0.2	1,4-Benzenedicarboxylic acid, diethyl ester	89
		2-(1-Cyclohexenyl)cyclohexanone	43
60.95	0.41	Cyclopentane, 1-butyl-2-propyl-	53
		Cyclohexane, 1,2,4-trimethyl-	50
		2,6,10-Dodecatrienal, 3,7,11-trimethyl-, (Z,E)-	35
61.95	0.59	[1,2,4]Triazolo[1,5-a]pyrazine	37
62.54	0.85	2(1H)-Pyridinone, 1-ethenyl-	45
		Dicyclopropylmethanol, pentafluoro propionate	38
		Cyclopropanecarbonitrile	35
62.65	0.65	9-Tricosene, (Z)-	99
		E-15-Heptadecenal	98
62.67	0.67	5-Eicosene, (E)-	99
		Cycloeicosane	98
		1-Octadecene	98
62.74	0.52	Octadecane	89
		Eicosane	86
63.07	1.03	1,5,9-Cyclododecatriene, 1,5,9-trimethyl-	38
63.48	0.92	3-Hexenedinitrile	81
		Spiro[bicyclo[2.2.1]hept-5-ene-2,1'-cyclopropane]	53
64.31	4.13	Pentanedinitrile, 2-methyl-	72
		2-Pentenenitrile	47
65.57	2.72	2-Pentenenitrile	52
		1,5-Cyclooctadiene, (E,Z)-	50
		Propargylamine	47
66.16	1.78	9-Tricosene, (Z)-	99
		1-Nonadecene	98

		Z-5-Nonadecene	95
68.18	0.1	Methyl 2-[1-(4-methylphenyl)hydrazino]propanoate	47
		3-Methyl-2,3-dihydro-benzo[b]thiophene-3-carboxylic acid, methyl ester	30
68.32	1.14	Butanenitrile, 2-methylene-	76
		Propanenitrile, 3-bromo-	50
		2-Pentenenitrile	45
68.51	0.16	1H-Indole, 1-methyl-2-phenyl-	35
69.66	1.9	9-Tricosene, (Z)-	99
		13-Tetradecen-1-ol acetate	93
71.44	0.44	n-Hexadecanoic acid	42
		2-(Acetoxymethyl)-3-(methoxycarbonyl)biphenylene	35
73.99	1.78	Octacosyl trifluoroacetate	91
		Hexadecane, 1-(ethenyloxy)-	89
		9-Tricosene, (Z)-	87
79.51	1.84	1-Nonadecene	92
		9-Tricosene, (Z)-	90
		Pyridine-3-carboxamide, oxime, N-(2-trifluoromethylphenyl)-	90
82.16	0.31	9-Tricosene, (Z)-	43
82.37	1.13	Hexatriacontyl pentafluoropropionate	70
		Tetratriacontyl pentafluoropropionate	70
		Octatriacontyl pentafluoropropionate	70
82.41	0.26	9-Tricosene, (Z)-	95
		11-Tricosene	93
82.47	0.88	Tetratriacontyl heptafluorobutyrat	64
		Tetratriacontyl trifluoroacetate	64
		Dotriacontyl trifluoroacetate	64
82.53	0.38	Hexadecane, 1-(ethenyloxy)-	93
		1-Decanol, 2-hexyl-	92
		Cyclopentadecane	91
82.58	0.45	Ethanol, 2-(tetradecyloxy)-	83
		1-Hentetracontanol	81
		Tetratriacontyl heptafluorobutyrat	76
82.64	1.37	13-Tetradecen-1-ol acetate	83
		Behenyl chloride	80
		1-Tricosene	70
84.16	0.97	N-Benzoylglycine ethyl ester	58
		S-Benzoyl-N-(p-methoxybenzylidene) thiohydroxylamine	50
		Hydrazinecarboxylic acid, 2-benzoyl-, ethyl ester	47
86.67	0.84	9-Tricosene, (Z)-	96
86.70	0.15	9-Tricosene, (Z)-	93
		Octatriacontyl pentafluoropropionate	50
		Decane, 5,6-bis(2,2-dimethylpropylidene)-, (Z,Z)-	46
86.72	0.64	1-Tricosene	94
		Eicosane	91
		Cyclohexadecane	84
95.95	0.19	Hexahydropyridine, 1-methyl-4-[4,5-dihydroxyphenyl]-	43

		Cyclotrisiloxane, hexamethyl-	27
		Acetamide, N-(4-bromo-2-chlorophenyl)-	25
96.13	1.92	9-Tricosene, (Z)-	72
		1-Nonadecene	70
		1-Decanol, 2-hexyl-	55
102.15	0.18	4-Cyanobenzophenone	35
102.18	0.02	1H-Indole, 1-methyl-2-phenyl-	35
108.61	0.38	1H-Indole, 5-methyl-2-phenyl-	35
108.66	0.11	9-Tricosene, (Z)-	90
		(+-)-cis-3,4-Dimethyl-2-phenyltetrahydro-1,4-thiazine	38
111.83	0.34	1-Nonadecene	92
		9-Tricosene, (Z)-	91
		17-Pentatriacontene	87
111.98	0.24	9-Tricosene, (Z)-	74
		Tetratriacontyl pentafluoropropionate	50
		Tetratriacontyl heptafluorobutyrat	50
112.01	0.04	9-Tricosene, (Z)-	86
		Oxalic acid, hexadecyl propyl este	45
112.05	0.07	Octatriacontyl pentafluoropropionate	50
		Hexatriacontyl pentafluoropropionate	45
		Octacosyl trifluoroacetate	42
112.07	0.04	Oxalic acid, hexadecyl propyl este	41
		Eicosane	38
		2-Thiopheneacetic acid, 6-ethyl-3-octyl ester	35
	0.9	Others	

**Table S4.** CS-F1@650 composition			
**RT**	**Area%**	**Library/ID**	**Qual**
6.984	1.53	Ethyl Acetate	86
9.293	1.81	Acetic acid	90
11.175	2.21	2-Propanone, 1-hydroxy-	45
26.345	1.04	Cyclohexanone	83
		2-Cyclopenten-1-one, 2-hydroxy-	80
		.alpha.-Pyrrolidone, N-methyl-5-bromomethyl-	72
27.920	0.17	2(5H)-Furanone	86
		Formamide, N-(cyanomethyl)-	43
27.935	0.27	2(5H)-Furanone	80
		2H-Pyran, 3,4-dihydro-	42
29.963	0.35	2,4-Diaminopyrimidine	64
		Resorcinol	58
		2-Amino-3-hydroxypyridine	53
32.055	0.99	2-Cyclopenten-1-one, 2-hydroxy-3-methyl-	94
32.452	0.42	Phenol	91
33.828	0.08	Acetophenone	87
34.907	0.26	l-Alanine, N-valeryl-, tetradecylester	45
		3-tert-Butyl-2-oxo-1,3-oxazolidine	43
		Oxalic acid, 4-chlorophenyl undecyl ester	43
35.466	0.34	p-Cresol	93
		Phenol, 2-methyl-	93
		Phenol, 3-methyl-	93
36.549	0.21	1-Butanone, 1-phenyl-	49
		Isopropyl phenyl ketone	49
		1,2-Propanedione, 1-phenyl-	47
36.613	0.43	Borinic acid, diethyl-, methyl ester	50
		Tetrahydrofurfuryl chloride	49
		Hexanoic acid, 2-tetrahydrofurylmethyl ester	40
36.830	0.37	Maltol	53
		4,5-Diamino-6-hydroxypyrimidine	50
36.928	1.07	p-Cresol	94
37.201	0.63	Cyclododecane	95
		1-Dodecene	93
37.430	0.38	Dodecane	95
		Tetradecane	74
		Pentadecane	72
38.086	0.99	Propanamide, N-methyl-2-amino-	45
		2-Formylhistamine	38
40.448	0.41	Benzene, 1,2-dimethoxy-	70
		1,3-Benzenediamine, 4-methoxy-	58
		Benzene, 1,4-dimethoxy-	58
41.355	15.62	Benzoic acid	96
41.928	0.8	E,Z-2,13-Octadecadien-1-ol	92
		Benzoic acid	60

42.097	1.07	1-Tridecene	97
		2-Tetradecene, (E)-	83
42.285	0.53	Tridecane	95
		Bacchotricuneatin c	59
42.926	1.08	2,2-Dimethyl-3-heptene trans	38
		Cyclohexane, 1,1,2-trimethyl-	38
43.330	0.51	8-Decen-2-one, 9-methyl-5-methylene	30
43.739	1.52	2-Acetylcyclopentanone	38
		Cyanamide, dibutyl-	38
44.811	0.26	Naphthalene, 1-methyl-	62
45.677	0.45	2-Furanacetic acid, .alpha.-hydroxy	30
45.823	1.15	Caprolactam	81
46.052	0.46	Benzoic acid, 4-methyl-	93
46.427	0.14	Bicyclo[3.1.1]heptan-3-one, 2,6,6-trimethyl-, (1.alpha.,2.beta.,5.alpha.)-	35
		Triallylsilane	35
		Cyclohexanone, 2-(1-mercapto-1-methylethyl)-5-methyl-, trans-	35
46.449	0.13	Bicyclo[3.1.1]heptan-3-one, 2,6,6-trimethyl-, (1.alpha.,2.beta.,5.alpha.)-	46
		Trichloroacetic acid, undec-10-enyl ester	43
		9-Undecenol, 2,10-dimethyl-	35
46.689	1.66	1-Tetradecene	99
46.850	0.71	Tetradecane	98
		Pentadecane	87
48.459	2.29	Biphenyl	94
48.860	0.37	Ninhydrin	53
		3-Pyridinecarbonitrile	49
		Phthalic anhydride	46
50.232	1.04	Methanol, oxo-, benzoate	91
		2-Chloroethyl benzoate	80
		Benzoic acid, silver(1+) salt	72
50.655	0.6	1,1'-Biphenyl, 2-methyl-	87
		Diphenylmethane	87
50.888	0.39	1,12-Tridecadiene	89
		Eicosen-1-ol, cis-9-	83
		Ethanol, 2-(9-octadecenyloxy)-, (Z)-	83
51.019	1.94	1-Pentadecene	99
51.158	0.86	Pentadecane	96
52.548	1	Cyclohexane, 1,2,4-trimethyl-	38
		Cyclohexane, 1-ethyl-2-propyl-	30
		9-Tricosene, (Z)-	30
52.979	0.41	Naphthalene, 1-(2-propenyl)-	70
		1,1'-Biphenyl, 2-methyl-	62
53.429	0.29	1,1'-Biphenyl, 3-methyl-	91
		1,1'-Biphenyl, 4-methyl-	87
53.527	0.3	Butylated Hydroxytoluene	91
		Phenol, 2,4,6-tris(1-methylethyl)-	90
55.000	0.38	Z,E-2,13-Octadecadien-1-ol	81

		cis-7-Tetradecen-1-ol	74
		11-Hexadecen-1-ol, acetate, (Z)-	70
55.109	1.74	Cetene	98
55.225	0.81	Hexadecane	98
		Tridecane, 1-iodo-	94
55.679	0.46	Cyclohexane, 1,1,3,5-tetramethyl-,trans-	41
		2,2-Dimethyl-3-heptene trans	38
		Cyclopropanol, 1-(3,7-dimethyl-1-octenyl)-	38
56.582	0.34	4-Methyl-2,6-dihydroxyquinoline	47
		7-Amino-4-methyl-1,8-naphthyridin-2-ol	47
57.808	0.71	Fluorene	94
58.378	0.32	Terephthalic acid, but-3-enyl ethyl ester	78
		Terephthalic acid, 2-chlorophenylethyl ester	59
		Terephthalic acid, ethyl 2-methylphenyl ester	59
58.539	0.02	Diethyl Phthalate	87
58.884	0.26	1,11-Dodecadiene	90
		1-Cyclohexylnonene	90
		1,19-Eicosadiene	74
58.974	1.75	3-Heptadecene, (Z)-	99
		E-14-Hexadecenal	99
		8-Heptadecene	98
59.075	0.79	Heptadecane	97
59.686	0.7	Benzene, 1,1'-(1,3-propanediyl)bis	93
59.974	0.6	Benzophenone	94
60.263	0.12	Benzaldehyde, 4,6-dimethoxy-2,3-dimethyl-	49
		Anthracene, 9-ethyl-9,10-dihydro-10-methyl-	35
60.953	0.71	1-Heptadecene	58
		Pyridine-3-carboxamide, oxime, N-(2-trifluoromethylphenyl)-	56
		Cyclohexane, 1,2,4-trimethyl-	50
61.976	0.62	5-Methyl-Z-5-docosene	74
		11-Tricosene	46
		9-Tricosene, (Z)-	41
62.231	3.27	Benzene, (3-nitropropyl)-	27
		1-Benzyl-1,2,3-triazole	27
		N-Benzyl-1H-benzimidazole	27
62.482	0.3	cis-Stilbene	90
		Stilbene	56
		Ethylene, 1,1-diphenyl-	50
62.658	3.55	E-15-Heptadecenal	99
		1-Octadecene	98
62.887	0.46	1,1-Diphenylcyclopropane	97
63.161	0.35	1-Cyclohexyl-1-(4-methylcyclohexyl)ethane	53
		Cyclohexane, 1-ethyl-2-propyl-	43
		Bicyclo[3.1.1]heptan-3-one, 2-(2,2-D292)-6,6-dimethyl-, (stereoisomer 2)	41
63.794	0.49	(2,4,6-Trimethylcyclohexyl) methanol	43
		Cyclohexane, 1,1,3,5-tetramethyl-,cis-	43

		Cyclohexanone, 2-(1-mercapto-1-methylethyl)-5-methyl-, trans-	38
64.870	0.41	9H-Fluoren-9-one	90
65.106	0.49	Benzene, 1,1'-(1,2-dimethyl-1,2-ethenediyl)bis-, (Z)-	55
		Benzene, 1,1'-(1,2-dimethyl-1,2-ethenediyl)bis-, (E)-	55
		5,6-Dimethyl-1,10-phenanthroline	51
66.152	3.62	1-Nonadecene	99
		3-Heptadecene, (Z)-	97
		Z-5-Nonadecene	95
68.263	0.5	7-(Butanoylamino)-4-chloro-3-(propyloxy)-1H-2-benzopyran-1-one	46
		4-Isothiazolol, 5-chloro-3-phenyl-	30
		4,7-Dichloro-3-methylquinoline	30
68.353	0.2	1H-Indene, 3-methyl-	35
		2-(Acetoxymethyl)-3-(methoxycarbonyl)biphenylene	35
		2,5-Diphenyl-1,5-hexadiene	25
68.506	0.47	2-Oxabicyclo[4.4.0]dec-9-en-8-one,1,3,7,7-tetramethyl-, (-)-(1R,3S,6R)-	20
69.462	0.34	Cyclohexane, 1-(cyclohexylmethyl)-4-ethyl-, trans-	42
69.668	3.12	9-Tricosene, (Z)-	99
		5-Eicosene, (E)-	97
69.905	0.43	Isophthalic acid, 2-chloroethyl ethyl ester	66
71.640	0.69	1,3-Dioxolane, 4-ethyl-5-octyl-2,2-bis(trifluoromethyl)-, trans-	70
		1-Cyclohexyl-1-(4-methylcyclohexyl)ethane	38
		Cyclopentane, 1-pentyl-2-propyl-	35
73.972	2.94	11-Tricosene	98
		9-Tricosene, (Z)-	91
		1-Heneicosanol	91
79.490	3.01	1-Nonadecene	96
		9-Tricosene, (Z)-	95
84.153	1.35	1,3-Propanediol dibenzoate	55
		1H-Indol-2-one, 2,3-dihydro-1-methyl-3,2'-spiro-(5-methyl-5-phenyl-1,3-dioxane)-	50
		N-Benzoylglycine ethyl ester	49
86.425	0.08	9-Tricosene, (Z)-	89
		Cyclohexane, 1,2-dimethyl-3-pentyl-4-propyl-	64
86.676	3.5	Cyclotetracosane	92
		1-Tricosene	86
		9-Tricosene, (Z)-	83
96.018	0.68	Cyclotetracosane	83
		9-Tricosene, (Z)-	78
		1-Tricosene	64
96.115	1.17	1-Tricosene	95
		1-Decanol, 2-hexyl-	91
		9-Tricosene, (Z)-	89
96.168	1.2	9-Tricosene, (Z)-	95
		11-Tricosene	92
		Tetracosyl heptafluorobutyrate	89
108.546	0.34	9-Tricosene, (Z)-	72
108.602	0.17	Fumaric acid, pent-4-en-2-yl tridecyl ester	38

108.632	0.12	9-Tricosene, (Z)-	90
110.701	1.48	(2,3-Diphenylcyclopropyl)methyl phenyl sulfoxide, trans-	50
110.746	0.58	1-benzylindole	38
		Azetidine, 1-benzyl-3,3-dimethyl-2-phenyl-	32
110.768	0.49	1-benzylindole	38
		Azetidine, 1-benzyl-3,3-dimethyl-2-phenyl-	32
110.806	2.91	1-benzylindole	23

**Table S5.** CS-F2@650 composition			
**RT**	**Area%**	**Library/ID**	**Qual**
6.586	0.08	Acetic acid, chloro-, isobutyl ester	50
		Cyclopentane, methyl-	43
		1-Pentene, 2-methyl-	43
6.987	0.58	Ethyl Acetate	90
9.3	2.42	Acetic acid	90
11.178	2	2-Propanone, 1-hydroxy-	59
14.335	0.14	Toluene	90
14.458	0.16	Propanoic acid	90
17.465	0.22	Cyclopentanone	90
17.731	0.8	2,4-Dimethyl-1-heptene	96
		2-Hexene, 3,5-dimethyl-	59
19.489	0.1	Pentanoic acid	42
		Butanoic acid, 4-chloro-	39
		Hexanoic acid	39
19.95	0.25	Ethylbenzene	91
		o-Xylene	87
20.61	0.65	Furfural	87
		3(2H)-Pyridazinone	64
22.087	5.31	Styrene	97
		Bicyclo[4.2.0]octa-1,3,5-triene	97
		Styrene	96
22.675	0.11	2-Furanmethanol	94
23.084	0.28	2-Propanone, 1-(acetyloxy)-	47
		2-Butanone, 3-methyl-	43
		2,3-Butanedione	43
24.46	0.15	2-Cyclopenten-1-one, 2-methyl-	91
25.015	0.24	Ethanone, 1-(2-furanyl)-	68
26.353	0.97	2-Cyclopenten-1-one, 2-hydroxy-	86
		Cyclopentanone, 2-methyl-	72
		1,3-Cyclopentanedione	72
26.439	0.51	1-Decene	91
		Cyclobutane, 1-butyl-2-ethyl-	72
26.75	0.38	Decane	58
27.241	0.53	.alpha.-Methylstyrene	93
27.706	0.41	Benzaldehyde	96
27.935	0.52	2(5H)-Furanone	86
		2-Butenal, 2-methyl-	47
28.426	0.68	2-Furancarboxaldehyde, 5-methyl-	95
29.082	0.27	2-Cyclopenten-1-one, 3-methyl-	96
29.775	0.33	Benzonitrile	94
		2-Ethynyl pyridine	91
29.951	0.49	1H-Imidazole-2-carboxaldehyde, 1-methyl-	59
		Imidazole, 1,4,5-trimethyl-	53
		Hydroquinone	50
30.581	0.1	Oxazolidine, 2-butyl-2-ethyl-3-methyl-	72
		Oxazolidine, 2,2-diethyl-3-methyl-	50
		Propanamide, N-(2,6-dimethylphenyl)-3-(4-morpholyl)-	47
31.016	0.16	But-3-en-1-ynyl methyl sulfide	53
		4-Methyl-5H-furan-2-one	49
		2-Pentenal, 2-methyl-	46
31.376	0.12	Indene	70
		Benzene, 1-propynyl-	64
31.477	0.44	3-Eicosene, (E)-	64
		Cyclopentane, propyl-	62
		Ethanone, 1-cyclopentyl-	53

31.702	0.37	4-Isopropyl-1,3-cyclohexanedione	64
		5-Ethyl-1-nonene	52
		Cyclooctane	38
32.024	1.32	3-Octene, (Z)-	80
		1,2-Cyclopentanedione, 3-methyl-	70
		2-Cyclopenten-1-one, 2-hydroxy-3-methyl-	62
32.238	0.34	Undecane	95
32.452	0.5	Phenol	94
32.636	0.15	6-Methyl-3-heptyne	76
		2-Cyclopenten-1-one, 2,3-dimethyl-	72
		Cyclooctene	58
33.831	0.62	Acetophenone	94
34.067	0.17	3-Hexene, 2,2,5,5-tetramethyl-, (Z)-	38
34.559	0.19	Benzoic acid, methyl ester	87
34.9	0.43	2,4,5-Trihydroxypyrimidine	38
		Butanal	35
35.143	0.54	Phenol, 2-methoxy-	93
35.462	0.49	Phenol, 2-methyl-	96
		p-Cresol	95
35.698	0.22	1H-Imidazole-4-carboxylic acid, methyl ester	64
		Furyl hydroxymethyl ketone	64
		Methyl 2-furoate	64
35.927	0.34	Benzene, (1-methylenebutyl)-	53
		2,3-Pyrazinedicarbonitrile, 5-amino-6-[(2,3-dihydro-3-hydroxy-2-oxo-1H-indol-3-yl)amino]-	38
		.alpha.-Methylstyrene	38
36.534	1.41	Isopropyl phenyl ketone	80
		1,2-Propanedione, 1-phenyl-	76
		Phenacyl thiocyanate	72
36.808	0.38	Maltol	94
36.931	0.89	Phenol, 3-methyl-	95
		p-Cresol	93
37.201	0.8	1-Dodecene	97
		Cyclododecane	94
37.426	0.54	Dodecane	94
38.086	0.54	Glutaraldehyde	42
		Cyclopropyl carbinol	42
		2-Butanamine, 3-methyl-	42
38.213	0.26	2-Hexynoic acid	60
		2H-Pyran-2-one	55
38.412	0.5	Benzoic acid, ethyl ester	94
38.783	0.12	1-Propanone, 1-phenyl-	64
		N(N'-Methyl-N'-nitroso(aminomethyl))benzamide	64
		1-Propanone, 1-phenyl-	64
39.387	0.22	Naphthalene	93
39.807	0.18	Phenol, 3,5-dimethyl-	81
		Phenol, 2,3-dimethyl-	81
40.122	0.11	Ethanone, 1-(4-methylphenyl)-	49
		o-Cymene	46
		Benzene, 1-methyl-3-(1-methylethyl)-	46
40.316	0.13	4H-Pyran-4-one, 3,5-dihydroxy-2-methyl-	92
		5-Hydroxy-2-methylthiopyrimidine	53
40.444	0.47	Creosol	98
		2-Methoxy-5-methylphenol	93
41.404	9.38	Benzoic acid	96
41.621	0.03	Benzoic acid	93
41.643	0.06	Benzoic acid	96
41.932	0.24	Benzoic acid	60
		1,12-Tridecadiene	46

		Z-12-Tetradecen-1-ol	46
42.09	0.75	1-Tridecene	99
		1-Pentadecene	93
42.285	0.4	Tridecane	95
42.926	1.49	Cyclohexane, 1,2,3-trimethyl-, (1.alpha.,2.beta.,3.alpha.)-	43
		Nonane, 2-methyl-3-methylene-	43
43.319	0.52	11-Methyldodecanol	52
		2-Undecanethiol, 2-methyl-	50
		Cyclopentane, (2-methylbutyl)-	43
43.728	1.87	Cyclohexane, 1,1-dimethyl-2-propyl	52
		Cyanamide, dibutyl-	41
		1-Hexene, 3,3-dimethyl-	38
44.567	0.16	5-Isopropyl-3,3-dimethyl-2-methylene-2,3-dihydrofuran	76
		Phenol, 4-ethyl-2-methoxy-	70
		O-Methoxy-.alpha.-methylbenzyl alcohol	58
44.819	0.24	Naphthalene, 1-methyl-	70
		Naphthalene, 2-methyl-	70
45.01	0.18	Cyclobutane, 1-butyl-2-ethyl-	49
		Citral	43
		2-Undecene, 7-methyl-	38
45.418	0.14	Valeric acid, 4-pentadecyl ester	35
45.67	1.26	5-Hydroxymethylfurfural	86
		2-Furancarboxylic acid, 5-(hydroxymethyl)-	38
45.842	1.19	Caprolactam	94
46.059	0.4	Benzoic acid, 4-methyl-	94
		Benzoic acid, 3-methyl-	81
46.438	0.24	7-Tetradecene, (E)-	38
		(2,4,6-Trimethylcyclohexyl) methanol	38
46.532	0.18	1,11-Dodecadiene	95
		1,12-Tridecadiene	89
		Cyclopropaneoctanoic acid, 2-[(2-pentylcyclopropyl)methyl]-, methylester	87
46.689	1.02	2-Tetradecene, (E)-	97
		1-Tetradecene	96
46.847	0.41	Tetradecane	98
		Hexacosane	90
47.21	0.19	4-Ethylbenzoic acid, 2-bromo-4-fluorophenyl ester	58
		4-Ethylbenzoic acid, 2,5-dichlorophenyl ester	53
		1-Propanone, 1-(2,4-dimethylphenyl)-2-methyl-	53
48.166	0.16	Benzoic acid, 2-(1-methylethyl)-	64
		1-Penten-3-one, 4-methyl-1-phenyl-	55
		Propanedioic acid, nitrile, hydrazide, N2-(1-oxo-3-phenyl-2-propenyl)-	49
48.455	1.67	Biphenyl	94
48.863	0.42	Indan-1,2,3-trione	72
		1,2-Benzenedicarboxylic acid	64
		[2,2'-Bi-1H-indene]-1,1'-dione, 2,2',3,3'-tetrahydro-2,2',3,3,3',3'-hexahydroxy-	64
50.22	0.84	2-Chloroethyl benzoate	93
		Benzoic acid, ethyl ester	70
50.509	0.06	3-Chloro-4-methoxytoluene	35
50.644	0.21	Diphenylmethane	89
50.88	0.13	1,13-Tetradecadiene	97
		9-Octadecen-1-ol, (Z)-	95
51.015	0.89	1-Pentadecene	99
51.154	0.44	Pentadecane	98
52.016	0.15	Vanillin	42
52.316	0.1	Ethyl 4-cyanobenzoate	64
		Benzoic acid, 4-cyano-	43
52.56	0.89	n-Propyl benzoate	38
		Acetophenone, 2-chloro-	35

52.968	0.17	1,1'-Biphenyl, 3-methyl-	70
		1,1'-Biphenyl, 2-methyl-	64
52.994	0.09	1,1'-Biphenyl, 2-methyl-	96
		1,1'-Biphenyl, 4-methyl-	93
53.418	0.21	1,1'-Biphenyl, 2-methyl-	96
		Diphenylmethane	93
53.665	0.21	Cyclopentane, 1-butyl-2-pentyl-	49
		Cyclohexane, 1,1,2-trimethyl-	46
		Tricosyl trifluoroacetate	46
54.085	0.23	1-Hexadecanethiol	50
		1H-Imidazole, 2-ethyl-4,5-dihydro-4-methyl-	41
		Cyclohexane, 1,1,2-trimethyl-	38
54.333	0.14	Phosphetane, 1-chloro-2,2,3,4,4-pentamethyl-, 1-oxide	42
54.546	0.2	Bibenzyl	53
		Acetic acid, [(phenylmethyl)thio]-	49
54.992	0.27	1,9-Tetradecadiene	91
		Z-13-Octadecen-1-yl acetate	70
		Cycloundecene, 1-methyl-	64
55.105	0.96	Cetene	98
55.225	0.54	Hexadecane	96
55.686	0.25	Bicyclo[3.1.1]heptan-3-one, 2,6,6-trimethyl-, (1.alpha.,2.beta.,5.alpha.)-	43
		Cyclohexane, 1,1,3,5-tetramethyl-,cis-	38
		1-Pentyn-3-ol, 3-methyl-, carbamat	35
56.578	0.38	4(3H)-Quinazolinone, 3-amino-2-methyl-	50
		4-Methyl-2,6-dihydroxyquinoline	49
		Precocene I	47
57.335	0.11	4,5-Decanediol, 6-ethyl-	41
		3-Hepten-2-one, 3-propyl-	38
		Silane, trifluoro(2-methyl-2-butenyl)-	38
57.362	0.07	Silane, trifluoro(2-methyl-2-butenyl)-	58
		Cyclopropanecarboxylic acid, methyl ester	47
		2-Heptenal, 2-propyl-	46
57.437	0.23	1-(4-methylthiophenyl)-2-propanone	64
		Homovanillic acid	58
		(-)-R-Phenethanamine, 1-methyl-N-vanillyl-	53
57.804	0.39	Fluorene	90
58.377	0.33	Terephthalic acid, 3,4-dichlorophenyl ethyl ester	72
		Isophthalic acid, ethyl 2-methoxyethyl ester	72
		Terephthalic acid, 3,5-difluorophenyl ethyl ester	72
58.542	0.07	Diethyl Phthalate	55
58.895	0.16	1,19-Eicosadiene	76
		1,9-Tetradecadiene	70
		Ethanol, 2-(9-octadecenyloxy)-, (Z)-	68
58.973	0.9	E-14-Hexadecenal	99
		3-Heptadecene, (Z)-	95
		1-Heptadecene	95
59.075	0.43	Heptadecane	98
59.311	0.07	Phenmethanol, 2,.alpha.,.alpha.-trimethyl-	49
		3,4-Dimethylbenzamide	46
		4-Ethylbenzoic acid, 2-chloroethyl ester	38
59.686	0.48	Benzene, 1,1'-(1,3-propanediyl)bis	97
59.974	0.51	Benzophenone	93
60.244	0.25	1,4-Benzenedicarboxylic acid, diethyl ester	46
		(2-Hydroxy-3,5-dimethylbenzoyl)formic acid	35
60.945	0.53	Cyclohexane, 1,2,4-trimethyl-	55
		Cyclohexane, 1,2,4,5-tetraethyl-, (1.alpha.,2.alpha.,4.alpha.,5.alpha.)-	52
		Cyclopentane, 1-pentyl-2-propyl-	49
61.965	0.44	Thieno[2,3-d]-1,3-thiaselenol-2-thione	56
		Cyclohexane, 1,2,4-trimethyl-	50

62.231	2.76	3-(Benzylthio)acrylic acid, methylester	35
62.651	2.4	E-15-Heptadecenal	99
		1-Nonadecene	94
		Trifluoroacetoxy hexadecane	94
62.883	0.32	1,1-Diphenylcyclopropane	95
		Naphthalene, 2-(1-cyclopenten-1-yl)-	93
		10,11-Dihydro-5H-dibenzo(a,d)cycloheptene	83
63.164	0.31	Cyclopentane, 1,1'-[3-(2-cyclopentylethyl)-1,5-pentanediyl]bis-	47
		Cyclohexane, 1,1,2-trimethyl-	38
		2,3,4-Trimethyl-hex-3-enal	30
63.476	0.27	Boranamine, 1-chloro-1-ethyl-N-methyl-N-phenyl-	42
63.794	0.37	Farnesol isomer a	38
		4-Hexen-1-ol, 2-ethenyl-2,5-dimethyl-	38
64.229	0.05	Phenol, 2-methyl-4-(1,1,3,3-tetramethylbutyl)-	47
		Phthalic acid, isohexyl neopentyl ester	38
		Phthalic acid, di(2-propylpentyl) ester	38
64.252	0.03	Pyridine-3-carbonitrile, 1-(2-hydroxypropyl)-4,6-dimethyl-2-oxo-1,2-dihydro-	46
		3',8,8'-Trimethoxy-3-piperidyl-2,2'-binaphthalene-1,1',4,4'-tetrone	46
		Diisooctyl phthalate	46
64.326	0.09	Bis(2-ethylhexyl) phthalate	53
		Phthalic acid, 6-ethyloct-3-yl 2-ethylhexyl ester	47
		Bis(2-ethylhexyl) phthalate	46
64.469	0.14	Phthalic acid, di(2-propylpentyl) ester	49
		4-Methoxyanthranilic acid	46
		2-(Nonyloxycarbonyl)benzoic acid	46
64.859	0.29	9H-Fluoren-9-one	93
		Benzo[h]cinnoline	87
65.099	0.23	Benzene, 1,1'-(3-methyl-1-propene-1,3-diyl)bis-	94
		Benzene, 1,1'-(1,2-dimethyl-1,2-ethenediyl)bis-, (E)-	92
65.815	0.15	Phenanthrene	92
		7,8-Diphenylbicyclo[4.2.1]nona-2,4,7-triene	89
		1,10b(2H)-Dihydropyrano[3,4,5-jk]fluorene	81
65.976	0.09	2-[4-Chlorophenyl]-5-pyrimidinamin	43
		Quinolin-5(6H)-one, 7,8-dihydro-2-hydroxy-4,7,7-trimethyl-	43
66.145	2.23	Z-5-Nonadecene	99
		1-Nonadecene	99
67.198	0.11	1,4-Benzenedicarboxylic acid, ethyl methyl ester	44
		Acetic acid, (2,3,4,5-tetrafluorophenyl)methyl ester	35
		1H-Indole, 5-methyl-2-phenyl-	25
68.266	0.75	Terephthalic acid, 2-chloroethyl isobutyl ester	38
		Succinic acid, di(4-chlorophenyl) ester	32
		Succinic acid, di(3-chlorophenyl) ester	32
68.502	0.26	Pyridine-3-carboxamide, oxime, N-(2-trifluoromethylphenyl)-	91
		1-Decanol, 2-hexyl-	43
		5-Methyl-Z-5-docosene	41
69.458	0.14	Cyclohexane, 1-(cyclohexylmethyl)-4-ethyl-, trans-	46
		Cyclohexane, 1,1'-(2-propyl-1,3-propanediyl)bis-	41
		Cyclohexane, 1,1'-(2-ethyl-1,3-propanediyl)bis-	38
69.473	0.06	Corydaldine	45
		2-Methyl-2-docosene	38
		1,3-Dioxolane, 4-ethyl-5-octyl-2,2-bis(trifluoromethyl)-, trans-	38
69.661	2.06	5-Eicosene, (E)-	98
		1-Octadecene	96
		9-Tricosene, (Z)-	96
69.904	0.31	Isophthalic acid, 2-chloroethyl ethyl ester	96
		(3R,5R,7aR)-3-Butyl-5-hexylhexahydro-1H-pyrrolizine	40
		Isophthalic acid	38
70.62	0.13	Trimethylsilyl 3-methylbenzoate	49

		Iminostilbene	46
		Glycine, N-[4-[(trimethylsilyl)oxy]benzoyl]-, methyl ester	46
71.426	0.63	Pentadecanoic acid	48
		n-Hexadecanoic acid	42
71.636	0.51	1-Cyclohexyl-1-(4-methylcyclohexyl)ethane	43
		Cyclohexane, 1-ethyl-2-propyl-	38
		Cyclohexanone, 2-(1-mercapto-1-methylethyl)-5-methyl-, trans-	35
73.975	2.1	11-Tricosene	97
		Octacosyl trifluoroacetate	91
		10-Heneicosene (c,t)	91
74.305	0.03	Cyclopentane, 1,1'-[3-(2-cyclopentylethyl)-1,5-pentanediyl]bis-	87
		Cyclohexane, 1,1'-(2-propyl-1,3-propanediyl)bis-	45
		But-2-enamide, N-decyl-N-methyl-3-methyl-	44
78.425	0.35	Bicyclo[3.1.1]heptan-3-one, 2-(2,2-dimethylpropyl)-6,6-dimethyl-, (stereoisomer 2)	50
		Fumaric acid, pent-4-en-2-yl tridecyl ester	32
		Ethyl 2-acetamido-3,3,3-trifluoro-2-(2-fluoroanilino)propionate	25
79.49	2.07	9-Tricosene, (Z)-	98
		1-Nonadecene	98
79.88	0.18	4-Dehydroxy-N-(4,5-methylenedioxy-2-nitrobenzylidene)tyramine	42
		Cyclopentane, 1,1'-[3-(2-cyclopentylethyl)-1,5-pentanediyl]bis-	38
82.155	0.02	p-Terphenyl	90
		m-Terphenyl	64
83.778	0.6	Cyclooctane, 1-methyl-3-propyl-	47
		Cyclohexane, 1,1,3,5-tetramethyl-,cis-	47
84.153	1.26	2-Benzoyl-3-isobutylidene-hexahydro-pyrrolo[1,2-a]pyrazin-1,4-dione	59
		4-Cyanobenzophenone	59
		Hydrazinecarboxylic acid, 2-benzoyl-, ethyl ester	52
85.979	0.27	Terephthalic acid, di(2-chloroethyl) ester	56
86.691	2.14	Octacosyl heptafluorobutyrate	93
		1-Tricosene	93
		Hexacosyl heptafluorobutyrate	93
96.134	1.87	Octacosyl heptafluorobutyrate	92
		Cyclotetracosane	90
		Dotriacontyl heptafluorobutyrate	86
108.56	0.7	1-Hexadecanol, 2-methyl-	83
		5-Eicosene, (E)-	78
		Hexadecane, 1-(ethenyloxy)-	62
108.613	0.93	9-Tricosene, (Z)-	81
		1-Decanol, 2-hexyl-	60
		9-Tricosene, (Z)-	55
110.81	5.25	(2,3-Diphenylcyclopropyl)methyl phenyl sulfoxide, trans-	38

**Table S6.** CS-F1@750 composition			
**RT**	**Area%**	**Library/ID**	**Qual**
5.394	0.86	N-(2,2-Dichloro-1-hydroxy-ethyl)-2,2-dimethyl-propionamide	50
		1,8-Nonadien-3-ol	38
		Acetic acid, chloro-, 1-methylbutyl ester	38
6.98	1.79	Ethyl Acetate	86
11.171	0.59	2-Propanone, 1-hydroxy-	59
18.458	0.3	Succindialdehyde	72
		N,N,N'-Trimethyl-1,3-propanediamin	40
		1,3-Propanediamine, N(1),N(1)-dimethyl-N(3)-(7-methyl[1,2,4]triazolo[4,3-a]pyrimidin-5-yl)-	38
22.664	0.76	2-Furanmethanol	97
26.346	1.04	2-Cyclopenten-1-one, 2-hydroxy-	80
		1,3-Cyclopentanedione	72
		Cyclopentanone, 2-methyl-	64
27.924	0.51	2(5H)-Furanone	59
		2H-Pyran, 3,4-dihydro-	43
32.047	1.57	2-Cyclopenten-1-one, 2-hydroxy-3-methyl-	91
		1,2-Cyclopentanedione, 3-methyl-	90
		2-Cyclopenten-1-one, 2-hydroxy-3-methyl-	87
32.452	1.21	Phenol	94
35.14	0.56	Phenol, 2-methoxy-	91
35.451	0.54	p-Cresol	95
		Phenol, 2-methyl-	94
36.609	0.66	Oxirane, butyl-	42
		3-Buten-2-ol, 2-methyl-	37
		1-Hydroxy-2-pentanone	36
36.92	2.14	p-Cresol	94
37.205	0.76	Cyclododecane	90
		1-Tridecene	83
		Cyclopentane, butyl-	76
37.43	0.45	Dodecane	95
38.086	1.93	Cyclopropyl carbinol	53
		Glutaraldehyde	36
		Pentanal	36
39.829	0.35	Phenol, 2,5-dimethyl-	93
		Phenol, 2-ethyl-	87
40.444	0.6	2-Methoxy-5-methylphenol	87
		Creosol	87
		2-Methoxy-6-methylphenol	83
41.243	0.9	Benzoic acid	93
41.34	0.08	Phenol, 3,5-dimethyl-	50
		Phenol, 3,4-dimethyl-	50
		Benzene, nitroso-	46
41.906	0.37	1,12-Tridecadiene	43
		Bicyclo[3.1.1]heptane, 2,6,6-trimethyl-, [1R-(1.alpha.,2.beta.,5.alpha.)]-	35
		(R)-(-)-(Z)-14-Methyl-8-hexadecen-1-ol	35
42.094	0.93	1-Tridecene	96
42.296	1.26	Tridecane	60
		Decane, 1-iodo-	35
42.933	0.47	Cyclohexane, 1,2,4-trimethyl-	38
43.664	1.71	3-Aminopiperidin-2-one	52
		Cyclopropanecarboxamide, N-2-methylpropyl	47
		dl-Citrulline	40
44.118	0.38	Phenol, 4-(1-methylethyl)-	87
		Phenol, 2-ethyl-6-methyl-	87
45.685	0.65	5-Hydroxymethylfurfural	58
		2,4-Hexadienedioic acid, (Z,Z)-	52
45.715	0.12	5-Hydroxymethylfurfural	43

		4-Pyrimidinol, 5-methoxy-	38
45.82	1.29	Caprolactam	76
		3,4-Dimethyl-isoxazol-5(4H)-one	50
46.528	0.44	9-Octadecen-1-ol, (Z)-	87
		cis-7-Dodecen-1-yl acetate	83
		9-Nonadecyne	76
46.689	1.58	Cyclotetradecane	95
		Cyclopropane, nonyl-	93
		1-Tetradecene	91
46.851	0.6	Tetradecane	94
48.477	0.61	Biphenyl	81
50.892	0.33	9-Octadecen-1-ol, (E)-	91
		11-Hexadecen-1-ol, (Z)-	87
		Cyclopentadecanol	86
51.019	1.93	1-Pentadecene	98
		1-Tridecene	97
51.158	0.86	Pentadecane	94
52.563	0.64	Phenol, 2-methoxy-4-(1-propenyl)-	92
		Phenol, 2-methoxy-5-(1-propenyl)-,(E)-	76
53.534	0.61	Butylated Hydroxytoluene	94
55	0.24	1-Hexadecyne	96
		1,13-Tetradecadiene	95
55.113	1.87	Z-8-Hexadecene	99
		Cetene	97
		Cyclohexadecane	94
55.233	1.04	Heneicosane	92
		Hexadecane	91
		Eicosane	89
55.367	0.2	trans-4-(2-(5-Nitro-2-furyl)vinyl)-2-quinolinamine	30
57.819	0.52	Fluorene	76
58.674	4.58	Cyclopentanecarboxamide, 3-ethenyl-2-(3-pentenylidene)-N-phenyl-, .alpha.,2Z(E),3.alpha.]-	59
58.88	0.55	Oxirane, hexadecyl-	60
		1,13-Tetradecadiene	58
		1-Cyclohexylheptene	55
58.981	2.34	E-14-Hexadecenal	99
		9-Nonadecene	95
		3-Heptadecene, (Z)-	95
59.082	0.95	Heptadecane	91
		Nonadecane, 9-methyl-	90
59.967	0.6	Benzophenone	94
60.451	0.7	N-Benzyl-N-ethyl-p-isopropylbenzamide	35
60.953	0.33	Pyridine-3-carboxamide, oxime, N-(2-trifluoromethylphenyl)-	92
		Cyclohexane, 1,1,2-trimethyl-	42
		Cyclohexane, 1-ethyl-2-propyl-	38
62.655	4.02	E-15-Heptadecenal	99
		1-Octadecene	98
63.806	0.36	Bicyclo[3.1.1]heptan-3-one, 2,6,6-trimethyl-, (1.alpha.,2.alpha.,5.alpha.)-	46
		Cyclohexane, 1-(cyclohexylmethyl)-4-ethyl-, cis-	45
66.16	4.49	Z-5-Nonadecene	99
		1-Heptadecene	97
		1-Nonadecene	95
69.669	4.49	9-Tricosene, (Z)-	98
		10-Heneicosene (c,t)	93
		Dotriacontyl pentafluoropropionate	93
71.652	0.36	Silicic acid, diethyl bis(trimethylsilyl) ester	38
		Pyridine-3-carboxamide, oxime, N-(2-trifluoromethylphenyl)-	30
		Fumaric acid, pent-4-en-2-yl tridecyl ester	27
73.987	4.23	1-Nonadecene	95

		Heptadecyl trifluoroacetate	93
		Heptadecyl heptafluorobutyrate	91
76.457	0.86	Piperidine, 1-(5-trifluoromethyl-2-pyridyl)-4-(1H-pyrrol-1-yl)-	56
		Formic acid, 1-(4,7-dihydro-2-methyl-7-oxopyrazolo[1,5-a]pyrimidin-5-yl)-, methyl ester	30
		4H-1,2,4-Triazole-3-thiol, 4-allyl-5-(1-naphthylmethyl)-	25
76.484	1.2	4H-1,2,4-Triazole-3-thiol, 4-allyl-5-(1-naphthylmethyl)-	30
		5-(p-Aminophenyl)-4-(p-tolyl)-2-thiazolamine	27
		3-Ethoxy-1,1,1,5,5,5-hexamethyl-3-(trimethylsiloxy)trisiloxane	27
77.394	1.14	N-Benzyl-N-ethyl-p-isopropylbenzamide	27
		2,2,2-Trifluoro-N-[2-(1-hydroxy-2,2,6,6-tetramethyl-piperidin-4-yl)-ethyl]-acetamide	15
		2,5-Dihydroxyacetophenone, bis(trimethylsilyl) ether	15
79.516	4.01	Ethanol, 2-(tetradecyloxy)-	93
		9-Tricosene, (Z)-	92
		1-Tricosene	91
83.842	1.6	3-Isopropoxy-1,1,1,5,5,5-hexamethyl-3-(trimethylsiloxy)trisiloxane	38
		2,2,2-Trifluoro-N-[2-(1-hydroxy-2,2,6,6-tetramethyl-piperidin-4-yl)-ethyl]-acetamide	35
83.902	1.13	5-(p-Aminophenyl)-4-(O-tolyl)-2-thiazolamine	30
		3,6-Bis(N-dimethylamino)-9-ethylcarbazole	30
86.687	4.09	9-Tricosene, (Z)-	95
		1-Tricosene	78
		Octatriacontyl pentafluoropropionate	78
91.249	1.21	Cyclotetrasiloxane, octamethyl-	30
		4H-1,2,4-Triazole-3-thiol, 4-allyl-5-(1-naphthylmethyl)-	25
91.287	0.32	Piperidine, 1-(5-trifluoromethyl-2-pyridyl)-4-(1H-pyrrol-1-yl)-	56
		1,2-Benzenediol, 3,5-bis(1,1-dimethylethyl)-	25
96.123	1.74	1-Tricosene	93
		Cyclotetracosane	92
		E-15-Heptadecenal	92
96.149	0.28	Ethanol, 2-(tetradecyloxy)-	91
		9-Tricosene, (Z)-	89
		13-Tetradecen-1-ol acetate	89
96.171	1.52	Ethanol, 2-(tetradecyloxy)-	91
		1-Tricosene	74
		Octacosyl trifluoroacetate	64
97.393	0.17	4-Nitro-4'-chlorodiphenylsulfoxide	53
97.461	0.05	Formic acid, 1-(4,7-dihydro-2-methyl-7-oxopyrazolo[1,5-a]pyrimidin-5-yl)-, methyl ester	30
		N-Benzyl-N-ethyl-p-isopropylbenzamide	27
104.733	0.34	3,6-Dioxa-2,4,5,7-tetrasilaoctane,2,2,4,4,5,5,7,7-octamethyl-	38
		3-Isopropoxy-1,1,1,5,5,5-hexamethyl-3-(trimethylsiloxy)trisiloxane	30
104.857	0.74	2-(Acetoxymethyl)-3-(methoxycarbonyl)biphenylene	25
		Ergonovine	25
		Formic acid, 1-(4,7-dihydro-2-methyl-7-oxopyrazolo[1,5-a]pyrimidin-5-yl)-, methyl ester	25
108.613	2.19	9-Tricosene, (Z)-	94
108.696	1.36	9-Tricosene, (Z)-	35
		Oxalic acid, hexadecyl propyl este	27
		Cyclohexane, 1,1'-(2-ethyl-1,3-propanediyl)bis-	27
112.189	2.47	5-(p-Aminophenyl)-4-(O-tolyl)-2-thiazolamine	30
		2,4-Cyclohexadien-1-one, 3,5-bis(1,1-dimethylethyl)-4-hydroxy-	20

		**Table S7.** Composition of CS-F2@750	
**RT**	**Area%**	**Library/ID**	**Qual**
5.398	0.29	Methylene chloride	76
6.582	0.14	Formic acid, 2-methylpentyl ester	28
6.991	0.7	Ethyl Acetate	80
9.308	3.35	Acetic acid	91
11.186	4.81	2-Propanone, 1-hydroxy-	45
14.335	0.09	Toluene	81
		Spiro[2,4]hepta-4,6-diene	81
		1,3,5-Cycloheptatriene	74
14.458	0.34	Propanoic acid	87
18.833	0.77	Propanoic acid, 2-oxo-, methyl ester	72
19.512	0.52	Butanoic acid	72
		Propanedioic acid, propyl-	39
20.591	0.99	Furfural	87
22.087	0.37	1,3,5,7-Cyclooctatetraene	96
		Styrene	94
		Bicyclo[4.2.0]octa-1,3,5-triene	81
22.653	2.33	2-Furanmethanol	98
23.076	0.5	2-Propanone, 1-(acetyloxy)-	72
		1,2-Ethanediol, diacetate	56
		2,3-Butanedione	50
23.406	0.07	Acetic acid, nitro-, methyl ester	38
		1-Cyclohexyl-3-ethoxy-butan-2-one	37
23.421	0.06	Hydrazine, 1,1-diethyl-	50
		Acetic acid, nitro-, methyl ester	33
		1,3-Dioxolane, 2-(1-bromoethyl)-	32
24.456	0.11	2-Cyclopenten-1-one, 2-methyl-	86
		Cyclopentene	70
24.482	0.07	2-Cyclopenten-1-one, 2-methyl-	80
		Cyclopentene	70
		2-Cyclopenten-1-one, 3-methyl-	59
25.011	0.29	Ethanone, 1-(2-furanyl)-	83
25.104	0.16	Crotonyl isothiocyanate	59
		Methacrylic anhydride	59
		2-Butenoyl chloride	59
26.349	2.39	2-Cyclopenten-1-one, 2-hydroxy-	86
		1,2-Cyclopentanedione	64
26.754	0.3	Propyl aldoxime, 2-methyl-, syn-	47
		Thiocyanic acid, 2-propynyl ester	47
		2-Furanmethanol	45
27.74	0.47	Butyrolactone	83
		Butanoic acid, 4-hydroxy-	64
27.931	1.27	2(5H)-Furanone	90
		Propargyl alcohol	43
28.415	1	2-Furancarboxaldehyde, 5-methyl-	94
29.082	0.68	1,3-Butadiene, 2-methyl-	86
		2-Cyclopenten-1-one, 2-methyl-	81
		2-Cyclopenten-1-one, 3-methyl-	70
29.757	0.14	Benzonitrile	87
		Tricyclo[3.1.0.0(2,4)]hex-3-ene-3-carbonitrile	86
		Benzonitrile	86
29.948	0.56	1H-Imidazole-2-carboxaldehyde, 1-methyl-	50
		4(1H)-Pyrimidinone, 6-methyl-	47
		1-(Dimethylamino)pyrrole	47

30.15	0.13	Tetrahydrofurfuryl chloride	53
		Borinic acid, diethyl-, methyl ester	53
		trans-2-Methyl-4-hexen-3-ol	50
30.574	0.26	Oxazolidine, 2,2-diethyl-3-methyl-	40
31.012	0.22	3-Buten-1-ol, 3-methyl-2-methylene	64
		Cyclopropanecarboxylic acid, 5-fluoro-2-nitrophenyl ester	59
		2-Propenoic acid, 2-methyl-, 2-propenyl ester	59
31.368	0.07	Indene	91
32.054	2.1	1,2-Cyclopentanedione, 3-methyl-	93
		2-Cyclopenten-1-one, 2-hydroxy-3-methyl-	91
32.231	0.18	Undecane	87
32.444	2.84	Phenol	94
32.647	0.33	2-Cyclopenten-1-one, 2,3-dimethyl-	81
		3-Methyl-3-cyclohexen-1-one	58
32.962	0.15	1-Oxetan-2-one, 4-methyl-3-methylene-	60
		4-Methyl-2,6,7-trioxa-1-phosphabicyclo[2.2.2]octane 1-oxide	50
		Cycloheptene	50
33.307	0.17	Benzene, 1-fluoro-4-methoxy-	50
		3H-Pyrazol-3-one, 1,2-dihydro-1,2,5-trimethyl-	50
		Benzene, 1-fluoro-2-methoxy-	50
33.839	0.28	Acetophenone	93
34.592	0.24	Phenol, 4-bromo-	68
		Phenol, 3-bromo-	62
		Phenol, 2-bromo-	62
34.907	0.57	2,4,5-Trihydroxypyrimidine	43
		1-Methyl-2,4,5-trioxoimidazolidine	42
		D-Alanine, N-allyloxycarbonyl-, heptadecyl ester	35
35.143	1.38	Phenol, 2-methoxy-	96
35.454	0.68	Phenol, 2-methyl-	98
		p-Cresol	94
35.694	0.31	Methyl 2-furoate	72
		Furan-2-carboxylic acid, 3-formylphenyl ester	64
36.017	0.31	Cyclopropane, 1,1-dichloro-2-methyl-2-(1-methylbutyl)-	47
		4-Methyl-5H-furan-2-one	38
36.628	1.62	3-Penten-2-ol	64
		2-Furanmethanol, tetrahydro-	64
		Tetrahydrofurfuryl acrylate	59
36.811	0.77	Maltol	91
36.916	1.83	p-Cresol	93
		Phenol, 2-methyl-	64
37.198	0.57	Cyclododecane	95
		Chloroacetic acid, undecyl ester	95
		1-Tridecene	91
37.419	0.47	Dodecane	87
		Tridecane	72
38.082	1.04	1,3-Propanediamine, N-methyl-	59
		Acetic acid, [(aminocarbonyl)amino]oxo-	42
		l-Guanidinosuccinimide	36
38.213	0.21	Bicyclo[3.1.0]hexan-3-one	53
		2-Cyclopenten-1-one, 3-methyl-	53
		2,3-Pentadiene	52
39.387	0.34	Naphthalene	93
		1H-Indene, 1-methylene-	87
		Azulene	87
39.822	0.33	Phenol, 2,5-dimethyl-	94

		Phenol, 2,3-dimethyl-	94
40.301	0.24	4H-Pyran-4-one, 3,5-dihydroxy-2-methyl-	76
		4H-Pyran-4-one, 3,5-dihydroxy-2-methyl-	58
		2,4(1H,3H)-Pyrimidinedione, dihydro-1,3-dimethyl-	47
40.436	0.98	Creosol	98
		2-Methoxy-5-methylphenol	93
41.254	1.72	Benzoic acid	97
41.88	0.3	Oxepane	46
		Cyclopentanone, 2-methyl-	43
		Cyclobutane, methyl-	43
41.898	0.18	Bicyclo[3.1.1]heptane, 2,6,6-trimethyl-, [1R-(1.alpha.,2.beta.,5.alpha.)]-	41
		Tridecanedial	38
		Propenal dimethylhydrazone	38
42.086	0.92	1-Tridecene	98
		Cyclodecane	91
42.3	1.5	Tridecane	38
		Heptane, 4-methyl-	35
42.922	0.95	Cyclohexane, 1,1,2-trimethyl-	38
		Cyclohexane, 1,1,3,5-tetramethyl-,trans-	38
		Octane, 4-methyl-	38
43.3	0.65	Oxalic acid, allyl pentadecyl este	43
43.66	2.67	1,4:3,6-Dianhydro-.alpha.-d-glucopyranose	87
		3-Aminopiperidin-2-one	52
		2-Dodecene, 4-methyl-	47
43.986	0.07	.alpha.-Irone	47
		Bicyclo[3.1.0]hexane, 6-isopropylidene-1-methyl-	46
		1,3-Cyclohexadiene, 1-methyl-4-(1-methylethyl)-	45
44.118	0.72	Phenol, 4-(1-methylethyl)-	93
44.564	0.4	Phenol, 4-ethyl-2-methoxy-	93
		Pyrazine, 2-methoxy-3-(1-methylethyl)-	81
44.811	0.25	Naphthalene, 2-methyl-	95
		Naphthalene, 1-methyl-	94
45.006	0.1	1-Cyclohexyl-2-methyl-prop-2-en-1-one	30
45.422	0.38	2-(Chloromethyl)tetrahydropyran	58
		2,6-Octadienal, 2,6-dimethyl-8-(tetrahydro-2H-2-pyranyloxy)	53
		(S)-(+)-2',3'-Dideoxyribonolactone	53
45.666	2.4	5-Hydroxymethylfurfural	89
		4-Fluorobenzyl alcohol	47
45.842	1.63	Caprolactam	94
		3,4-Dimethyl-5-hydroxy-isoxazole	60
46.442	0.14	Bicyclo[3.1.1]heptan-3-one, 2,6,6-trimethyl-, (1.alpha.,2.beta.,5.alpha.)-	50
		Cyclopentanone, 3-methyl-2-(2-pentenyl)-	49
46.532	0.3	9-Octadecen-1-ol, (E)-	91
		1,13-Tetradecadiene	64
		9-Decen-1-ol, trifluoroacetate	58
46.678	1.68	1-Tetradecene	93
		9-Octadecene, (E)-	55
		Decyl trifluoroacetate	55
46.85	0.47	Tetradecane	97
48.177	0.4	3-Allyl-6-methoxyphenol	95
		Eugenol	93
48.459	0.69	Biphenyl	86
48.875	0.42	Phthalic anhydride	38
		3-(Phthalimidomethyl)benzoic acid	35
49.778	0.26	Phenol, 2,6-dibromo-	84

		Naphthalene, 1,6-dimethyl-	55
		Phenol, 2,4-dibromo-	53
50.232	0.32	Hydrazine, 1-methyl-1-phenyl-	41
		Benzoic acid	41
		1,2-Ethanediol, monobenzoate	38
50.367	0.06	Benzaldehyde, 2,5-dimethoxy-	35
		2-Furancarboxylic acid, 1-methylethyl ester	30
50.52	0.41	3-Allyl-6-methoxyphenol	60
		Phenol, 2-methoxy-3-(2-propenyl)-	60
		Phenol, 2-methoxy-4-(1-propenyl)-	55
50.655	0.28	1,1'-Biphenyl, 2-methyl-	78
50.876	0.26	1-Cyclohexylheptene	86
		Z-13-Octadecen-1-yl acetate	78
		7-Pentadecyne	78
51.011	1.18	1-Pentadecene	98
		1-Tridecene	95
		5-Octadecene, (E)-	91
51.158	0.74	Eicosane	97
		Pentadecane	96
52.016	0.42	Vanillin	97
		Benzaldehyde, 3-hydroxy-4-methoxy-	76
52.563	1.24	Phenol, 2-methoxy-4-(1-propenyl)-	98
		trans-Isoeugenol	97
		Phenol, 2-methoxy-4-(1-propenyl)-,(Z)-	96
53.654	0.19	Borane, 2,3-dimethyl-2-butyl- (dimer)	46
		9-Undecen-2-one, 6,10-dimethyl-	41
		5,9-Undecadien-2-one, 6,10-dimethyl-, (Z)-	35
54.303	0.16	1-[1-Methyl-2-(methyloxy)ethyl]-1-phenylhydrazine	25
		Benzaldehyde, 3-methoxy-	25
54.989	0.27	1,12-Tridecadiene	93
		5-Nonadecen-1-ol	90
		Bicyclo[3.3.2]decan-9-one	89
55.105	1.02	Cetene	98
		Cyclohexadecane	96
55.225	0.68	Hexadecane	98
55.547	0.16	Ethanone, 1-(3-hydroxy-4-methoxyphenyl)-	90
		Apocynin	90
		1,4-Dimethoxy-2,3-dimethylbenzene	87
55.686	0.12	1-Cyclohexyl-2-methyl-prop-2-en-1-one	53
		6,11-Dimethyl-2,6,10-dodecatrien-1-ol	50
		3,7-Nonadien-2-one, 4,8-dimethyl-	49
57.459	0.52	Phenol, 2-methoxy-4-propyl-	81
		Homovanillyl alcohol	74
57.718	0.15	3-Methyl-4-nitrobenzoic acid	44
		4,5,6,6a-Tetrahydro-2(1H)-pentalenone	41
57.744	0.07	4(5H)-Benzofuranone, 6,7-dihydro-3,6-dimethyl-, (R)-	45
		1,6-Dimethyl-5-oxo-1,2,3,5-tetrahydroimidazo[1,2-a]pyrimidine	42
		N-(1-Cyclohexen-1-yl)piperidine	38
57.778	0.21	Fluorene	53
58.441	0.21	3,4,5-Trimethyl-1H-pyrano[2,3-c]pyrazol-6-one	95
		2,4-Dimethyl-3-acetyl-5-formylpyrrole	46
		1,1-Dichloro-2-methyl-3-(4,4-diformyl-1,3-butadien-1-yl)cyclopropane	43
58.535	0.21	2-Naphthalenol	64
		1-Naphthalenol, acetate	49
58.576	0.04	Chloroacetic acid, 1-naphthyl este	55

		2-Naphthyl carbamate	49
		Furan, 3-phenyl-	46
58.651	0.37	Ethyl .alpha.-d-glucopyranoside	49
		d-Gluco-heptulosan	46
		Acetamide, N-(4-hydroxycyclohexyl)-, trans-	46
58.876	0.3	2(1H)-Benzocyclooctenone, decahydro-10a-methyl-, trans-	89
		E-10-Methyl-11-tetradecen-1-ol propionate	83
		Ethanol, 2-(9-octadecenyloxy)-, (Z)-	81
58.973	1.1	9-Tricosene, (Z)-	99
		E-14-Hexadecenal	95
		3-Eicosene, (E)-	91
59.075	0.69	Heptadecane	96
		Octadecane	94
59.393	0.49	Octadecanoic acid, 2-hydroxy-1,3-propanediyl ester	27
59.697	0.36	Benzene, 1,1'-(1,3-propanediyl)bis	95
59.974	0.27	Benzophenone	81
60.259	0.17	Methyl piperonylate, 2-hydroxy-	40
		7-Hydroxycoumarin	38
		Naphthalene, 1,2,3,4-tetrahydro-6-methoxy-	35
60.956	0.48	Pyridine-3-carboxamide, oxime, N-(2-trifluoromethylphenyl)-	91
		Cyclopentane, 1-pentyl-2-propyl-	78
		1,3-Dioxolane, 4-ethyl-5-octyl-2,2-bis(trifluoromethyl)-, trans-	50
61.98	0.37	1-Hexadecanol, 2-methyl-	45
		(E)-pent-2-en-3-yl hexanoate	38
		Cyclohexane, 1,1,4,4-tetramethyl-	30
62.235	1.06	3-Benzyl-5-chloro-1,2,3-triazole 1-oxide	60
62.651	1.68	1-Octadecene	99
		1-Nonadecene	94
62.722	0.59	Octadecane	95
		Eicosane	90
63.164	0.37	Tridecane, 6-propyl-	78
		Nonadecane	47
		Nonane, 4,5-dimethyl-	46
63.479	0.24	Phenol, 3-phenoxy-	47
		Thiazolo[5,4-f]quinoline	43
		2-Trifluoromethyl-1H-benzoimidazol	38
63.79	0.38	11,13-Dimethyl-12-tetradecen-1-olacetate	37
		Cyclohexanone, 2-(1-mercapto-1-methylethyl)-5-methyl-, trans-	35
		1-Formyl-2,2-dimethyl-3-trans-(3-methyl-but-2-enyl)-6-methylidene-cyclohexane	32
64.24	0.33	Benzofuran-2-one, 2,3-dihydro-3,3-dimethyl-4-nitro-	25
64.484	0.2	Adenosine, N-methyl-	38
65.117	0.08	Benzene, 1,1'-(1-butenylidene)bis-	52
		1-Phenylprop-1-ene (1-3)sultine	44
		Benzeneacetaldehyde, .alpha.-(phenylmethylene)-	42
65.414	0.25	3-Hydroxybiphenyl	70
		p-Hydroxybiphenyl	70
65.785	0.24	5-Chloro-4-methyl-8-quinolinemethanol	53
		1-Benzazirene-1-carboxylic acid, 2,2,5a-trimethyl-1a-[3-oxo-1-butenyl] perhydro-, methyl ester	41
		7-Chlorocinchoninic acid	35
66.16	2.41	9-Tricosene, (Z)-	99
		1-Nonadecene	98
66.711	0.03	3-Methylindole-2-carboxylic acid,4,5,6,7-tetrahydro-, ethyl ester	25
		1-Butyldimethylsilyloxy-2-isopropoxybenzene	25
		Acetamide, 2-(2,4-dimethoxybenzylidenehydrazino)-N-ethyl-2-oxo-	25
68.169	0.11	Hexadecanoic acid, methyl ester	72

68.51	0.47	Cyclohexane, 1-(cyclohexylmethyl)-4-ethyl-, cis-	58
		Fumaric acid, 3-fluorophenyl hexadecyl ester	52
		Cyclohexane, 1-(cyclohexylmethyl)-4-ethyl-, trans-	46
69.462	0.13	5-Methyl-Z-5-docosene	49
		2-Butenenitrile, 2-chloro-3-(4-methoxyphenyl)-	44
		Thiocarbamic acid, N,N-dimethyl, S-1,3-diphenyl-2-butenyl ester	43
69.507	0.01	1H-Pyrrolo[2,3-b]pyridine-1-propionitrile, 2-phenyl-	35
		Benzo[h]quinoline, 2,4-dimethyl-	35
69.672	2.08	11-Tricosene	99
		1-Octadecene	98
		9-Tricosene, (Z)-	98
71.445	1.61	n-Hexadecanoic acid	97
		Tridecanoic acid	95
		n-Hexadecanoic acid	94
71.61	0.38	1,3-Dioxolane, 4-ethyl-5-octyl-2,2-bis(trifluoromethyl)-, trans-	45
		2-Methyl-5,5-diphenyl-4-(methylthio)imidazole	44
		Fumaric acid, pent-4-en-2-yl tridecyl ester	37
73.983	2.19	11-Tricosene	95
		Z-5-Nonadecene	95
		9-Tricosene, (Z)-	93
78.421	0.04	2-(Acetoxymethyl)-3-(methoxycarbonyl)biphenylene	35
		Benzene, 1-phenyl-4-(2-cyano-2-phenylethenyl)	35
79.497	2.57	1-Docosene	97
		9-Tricosene, (Z)-	95
79.88	0.27	Acetic acid, [4-(1,1-dimethylethyl)phenoxy]-, methyl ester	35
81.375	0.72	9-Octadecenoic acid, (E)-	55
		Dodecahydropyrido[1,2-b]isoquinolin-6-one	46
		tert-Butyl(5-isopropyl-2-methylphenoxy)dimethylsilane	38
81.566	0.02	[1,2,4]Triazolo[1,5-a]pyrimidine-6-carboxylic acid, 4,7-dihydro-7-imino-, ethyl ester	35
		Benzene, 2-[(tert-butyldimethylsilyl)oxy]-1-isopropyl-4-methyl-	30
81.6	0.01	1,2-Benzisothiazol-3-amine tbdms	42
		Purine-2,6-dione, 8-(3-ethoxypropylamino)-1,3-dimethyl-3,9-dihydro-	38
		5-Methyl-2-phenylindolizine	30
82.474	0.07	9-Tricosene, (Z)-	59
		Hexadecane, 1-(ethenyloxy)-	56
		13-Tetradecen-1-ol acetate	55
82.526	0.07	9-Tricosene, (Z)-	90
		4-Quinolinecarboxylic acid, 2-chloro-	47
		Eicosane	43
82.556	0.04	cis-Inositol tri-methylboronate	45
		5-Octadecene, (E)-	25
		Pyrido[2,3-d]pyrimidine, 4-phenyl-	25
83.786	0.18	Indolizine, 2-(4-methylphenyl)-	38
		5-Methyl-2-phenylindolizine	35
		Benzo[h]quinoline, 2,4-dimethyl-	35
83.801	0.12	1H-Indole, 5-methyl-2-phenyl-	30
		1,2,4-Triazol-3-amine, 5-(1,3,5-trimethyl-4-pyrazolyl)amino-	30
86.665	0.96	1-Nonadecene	90
		1-Heneicosyl formate	89
		Hexadecane, 1-(ethenyloxy)-	89
86.695	1.24	1-Tricosene	92
		9-Tricosene, (Z)-	91
		Dotriacontyl heptafluorobutyrate	90
94.81	0.01	Phenol, 4,4'-(1-methylethylidene)bis-	95
94.878	0.04	Phenol, 4,4'-(1-methylethylidene)bis-	95

		2-Trimethylsilyl-3-trimethylsilylamino-1,2,4-triazole	60
94.938	0.06	Phenol, 4,4'-(1-methylethylidene)bis-	92
94.979	0.03	Phenol, 4,4'-(1-methylethylidene)bis-	64
96.115	2.29	9-Tricosene, (Z)-	96
		Cyclotetracosane	96
		1-Nonadecene	96
108.594	0.7	Octatriacontyl pentafluoropropionate	90
		13-Tetradecen-1-ol acetate	83
		1-Hexadecanol, 2-methyl-	78
108.677	0.09	E-15-Heptadecenal	74
		Eicosane	70
		9-Tricosene, (Z)-	60
108.718	0.12	9-Tricosene, (Z)-	93
110.795	0.17	(2,3-Diphenylcyclopropyl)methyl phenyl sulfoxide, trans-	37
		1-benzylindole	32
		Azetidine, 1-benzyl-3,3-dimethyl-2-phenyl-	17

**Table S8.** Composition of Tar-F1			
**RT**	**Area%**	**Library/ID**	**Qual**
5.998	0.53	Furan, 2-methyl-	93
21.809	0.44	Styrene	83
		Bicyclo[4.2.0]octa-1,3,5-triene	64
31.069	0.89	Benzene, 1-ethynyl-4-methyl-	91
		Indene	90
		Benzene, 1-propynyl-	90
39.076	11.39	Naphthalene	95
		Azulene	94
44.5	3.11	Naphthalene, 2-methyl-	97
		Naphthalene, 1-methyl-	95
45.321	3.07	Naphthalene, 1-methyl-	91
		1,4-Methanonaphthalene, 1,4-dihydro	91
		Naphthalene, 2-methyl-	91
48.151	9.54	Biphenyl	95
49.887	1.64	Acenaphthene	59
		Biphenyl	55
50.352	0.93	Naphthalene, 1-(2-propenyl)-	89
		1,1'-Biphenyl, 2-methyl-	89
		1,1'-Biphenyl, 4-methyl-	76
50.801	3.97	Naphthalene, 2-ethenyl-	96
		Biphenyl	87
52.128	10.72	Biphenylene	93
		Acenaphthylene	90
52.702	1.03	1,1'-Biphenyl, 3-methyl-	87
		1,1'-Biphenyl, 3-methyl-	87
53.129	0.76	1,1'-Biphenyl, 4-methyl-	83
		Naphthalene, 1-(2-propenyl)-	76
		1,1'-Biphenyl, 2-methyl-	64
53.182	0.3	Acenaphthene	64
		1,4-Ethenonaphthalene, 1,4-dihydro	58
		Benzene, (2,4-cyclopentadien-1-ylidenemethyl)-	49
53.197	0.55	Acenaphthene	76
		Naphthalene, 2-ethenyl-	62
54.67	0.35	Dibenzofuran	64
		3,5-Dimethoxybenzyl alcohol	64
54.689	0.37	Dibenzofuran	72
		1(2H)-Acenaphthylenone	53
55.18	0.76	1-Naphthalenecarbonitrile	87
		2-Naphthalenecarbonitrile	83
56.923	0.48	9H-Fluorene, 9-(1-methylethyl)-	83
		Fluorene-9-methanol	78
		9H-Fluorene-9-carboxylic acid	72
56.938	0.6	1H-Phenalene	83

		9H-Fluorene, 9-(1-methylethyl)-	78
		Naphthalene, 1-(2-nitro-2-propenyl)	64
57.493	6.78	Fluorene	91
		Fluorene-9-methanol	78
57.826	0.22	1H-Phenalene	64
		9H-Fluorene-9-carboxylic acid	56
		Fluorene-9-methanol	56
57.849	0.16	Methyl ortho-O-aminobenzoate	91
		9H-Fluorene, 9-(1-methylethyl)-	53
		5-Fluoro-3-methyl-1-benzothiophene	52
57.916	1.06	9H-Fluorene, 1-methyl-	70
58.868	0.96	(E)-Stilbene	60
		9H-Fluorene, 9-methyl-	60
		1,1'-Biphenyl, 4-ethenyl-	55
59.36	0.67	Fluorene	87
		Benzaldehyde, 2,4-dihydroxy-3,6-dimethyl-	72
59.382	0.99	Fluorene	90
		Fluorene-9-methanol	86
59.689	0.73	Benzophenone	90
61.556	0.73	Anthracene, 1,2-dihydro-	93
		Anthracene, 9,10-dihydro-	86
		Phenanthrene, 9,10-dihydro-	86
62.179	0.6	Phenanthrene, 9,10-dihydro-	86
		Stilbene	86
		Anthracene, 9,10-dihydro-	83
62.194	0.59	(E)-Stilbene	95
64.105	0.02	Naphtho[2,3-b]norbornadiene	86
		Anthracene, 1-methyl-	70
		Phenanthrene, 2-methyl-	64
65.519	18.2	Anthracene	95
		Phenanthrene	95
65.875	3.69	Phenanthrene	95
		2-Cyclopropen-1-one, 2,3-diphenyl-	93
		Anthracene	93
67.348	1.87	Naphthalene, 1-phenyl-	89
		1,4-Ethenoanthracene, 1,4-dihydro-	89
		Dibenzo[a,e]cyclooctene	86
68.068	0.76	1H-Indene, 2-phenyl-	91
		1H-Cyclopropa[l]phenanthrene,1a,9b-dihydro-	91
69.65	0.83	Phenanthrene, 2-methyl-	89
		Anthracene, 2-methyl-	86
		Phenanthrene, 1-methyl-	64
69.886	0.37	Phenanthrene, 9-methyl-	55
		Anthracene, 9-methyl-	55
		1H-Indene, 2-phenyl-	55

69.912	0.06	Phenanthrene, 2-methyl-	50
		Phenanthrene, 1-methyl-	50
		Anthracene, 9-methyl-	50
70.632	0.93	6H-Cyclobuta[jk]phenanthrene	64
		4H-Cyclopenta[def]phenanthrene	59
70.654	0.4	Benzaldehyde, 3,5-dichloro-2-hydroxy	58
		4H-Cyclopenta[def]phenanthrene	53
		6H-Cyclobuta[jk]phenanthrene	53
72.472	2.1	Naphthalene, 2-phenyl-	89
72.487	2	Naphthalene, 2-phenyl-	95
78.331	1.46	Fluoranthene	86
		Pyrene	76
78.354	1.32	Fluoranthene	89
		Pyrene	83
79.793	0.3	Fluoranthene	86
		Benzene, 1,1'-(1,3-butadiyne-1,4-diyl)bis-	70
79.82	0.17	Fluoranthene	95
		Pyrene	83
81.552	0.17	Pyrene	55
		1,4-Pentadiyn-3-one, 1,5-diphenyl-	53
81.589	0.23	Fluoranthene	70
		Pyrene	60
81.619	0.21	Pyrene	92
		Fluoranthene	64
81.656	0.07	Fluoranthene	94
		Pyrene	70

Table S9. Composition of Tar-F2

RT	Area%	Library/ID	Qual
5.971	0.26	Furan, 2-methyl-	81
		Furan, 3-methyl-	64
8.502	1.85	Benzene	94
14.076	0.37	Toluene	81
21.798	0.77	Styrene	97
		Bicyclo[4.2.0]octa-1,3,5-triene	96
27.354	0.01	Isobutyronitrile	37
		4-Aminopyrimidine	25
31.08	0.93	Indene	97
		Benzene, 1-propynyl-	94
32.193	0.34	Phosphonic acid, (p-hydroxyphenyl)	90
		Benzene, ethoxy-	74
		Acetic acid, phenyl ester	74
36.62	0.13	4-Piperidinone, 2,2,6,6-tetramethyl-	47
		4,4-Dimethyl-2-imidazoline	22
36.658	0.04	1H-Indene, 1-methyl-	50
		1H-Indene, 3-methyl-	46
37.674	0.01	Naphthalene	90
		Azulene	81
39.072	11.8	Naphthalene	95
		Azulene	91
43.829	0.03	1H-Indene, 1-ethylidene-	87
		1,4-Methanonaphthalene, 1,4-dihydro	87
		Benzocycloheptatriene	87
44.492	3.06	Naphthalene, 1-methyl-	91
		1,4-Methanonaphthalene, 1,4-dihydro	91
45.306	2.8	Naphthalene, 2-methyl-	96
		Naphthalene, 1-methyl-	94
48.155	8.66	Biphenyl	95
49.887	1.32	.beta.-(1-Naphthyl)acrylic acid	64
		1-Isopropenylnaphthalene	64
		Acenaphthene	49
50.183	0.38	Naphthalene, 1,2-dimethyl-	94
		Naphthalene, 1,4-dimethyl-	93
		Naphthalene, 2,3-dimethyl-	93
50.34	0.41	Diphenylmethane	95
		1,1'-Biphenyl, 3-methyl-	70
50.355	0.37	Diphenylmethane	93
		1,1'-Biphenyl, 2-methyl-	86
50.798	3.4	Naphthalene, 2-ethenyl-	95
		Biphenyl	76
52.128	8.62	Biphenylene	94
		Acenaphthylene	91
52.698	0.9	1,1'-Biphenyl, 2-methyl-	93

		1,1'-Biphenyl, 3-methyl-	93
53.148	1.44	1,1'-Biphenyl, 2-methyl-	74
		Naphthalene, 2-(1-methylethenyl)-	62
		Naphthalene, 1-(2-propenyl)-	59
54.678	0.44	Benzenemethanol, 3,4-dimethoxy-	72
		Dibenzofuran	64
		3,5-Dimethoxybenzyl alcohol	64
55.18	0.59	2-Naphthalenecarbonitrile	90
56.559	0.2	1-Naphthalenecarbonitrile	97
		2-Naphthalenecarbonitrile	91
56.571	0.16	1-Naphthalenecarbonitrile	83
		2-Naphthalenecarbonitrile	72
56.93	0.84	9H-Fluorene, 9-(1-methylethyl)-	64
		1H-Phenalene	64
		Fluorene-9-methanol	59
57.481	2.1	Fluorene	81
57.493	2.92	Fluorene	94
		Fluorene-9-methanol	78
57.781	0.06	Methanesulfonic acid, 7,8,9,10-tetrahydrocyclohepta[de]naphthalen-8-yl ester	32
		Diphenylacetyl chloride	32
		9,10-Anthracenedione, 1-methyl-	32
57.909	1.2	1,1'-Biphenyl, 4-ethenyl-	70
		(E)-Stilbene	70
58.876	0.6	cis-Stilbene	58
		3H-Benz[e]indene, 2-methyl-	52
		(E)-Stilbene	52
58.895	0.36	9H-Fluorene, 9-methyl-	62
		(E)-Stilbene	60
		9H-Fluorene, 2-methyl-	53
59.352	1.2	Fluorene	93
59.674	0.59	Benzophenone	94
61.56	0.54	Phenanthrene, 9,10-dihydro-	95
		Phenanthrene, 1,2-dihydro-	94
61.807	0.31	9H-Fluorene, 1-methyl-	90
61.927	0.36	9H-Fluorene, 1-methyl-	89
		9H-Fluorene, 2-methyl-	89
62.179	1.03	Stilbene	91
		Anthracene, 9,10-dihydro-	83
		cis-Stilbene	83
62.291	0.04	4a,9a-Methano-9H-fluorene	42
		9H-Fluorene, 2-methyl-	25
		9H-Fluorene, 9-methyl-	25
62.595	0.38	9H-Fluorene, 2-methyl-	42
		9H-Fluorene, 1-methyl-	42
		Anthracene, 1,2-dihydro-	41
63.494	0.55	7,8-Diphenylbicyclo[4.2.1]nona-2,4 1,7-triene	81

		1,10b(2H)-Dihydropyrano[3,4,5-jk]fluorene	76
		Diphenylacetylene	70
64.098	0.19	Anthracene, 9-methyl-	78
		1H-Indene, 2-phenyl-	78
		Anthracene, 1-methyl-	60
64.117	0.27	1H-Cyclopropa[l]phenanthrene,1a,9b-dihydro-	90
		1H-Indene, 2-phenyl-	83
64.559	0.48	9H-Fluoren-9-one	95
65.507	13.76	Anthracene	94
		Phenanthrene	93
65.878	3.08	Anthracene	95
		Phenanthrene	93
66.017	0.06	9,10-Methanoanthracen-11-ol, 9,10-dihydro-9,10,11-trimethyl-	49
		Phenanthrene, 2-methyl-	46
		5H-Dibenzo[a,d]cycloheptene	46
67.352	1.76	Anthracene, 9-ethenyl-	86
		Cyclobuta[1",2":3,4:3",4":3',4']dicyclobuta[1,2:1',2']dibenzene,4b,4c,8b,8c-tetrahydro-	86
		Dibenzo[a,e]cyclooctene	86
68.056	0.68	1H-Indene, 2-phenyl-	83
		Phenanthrene, 2-methyl-	64
		Phenanthrene, 4-methyl-	60
69.65	0.39	Anthracene, 2-methyl-	95
		Anthracene, 1-methyl-	93
		1H-Cyclopropa[l]phenanthrene,1a,9b-dihydro-	92
69.672	0.32	Anthracene, 1-methyl-	60
		Phenanthrene, 2-methyl-	50
69.878	0.5	Phenanthrene, 1-methyl-	95
		Anthracene, 2-methyl-	92
		Phenanthrene, 2-methyl-	92
69.908	0.39	Phenanthrene, 2-methyl-	83
		Anthracene, 9-methyl-	83
70.635	1.73	4H-Cyclopenta[def]phenanthrene	58
		6H-Cyclobuta[jk]phenanthrene	50
70.755	0.21	Anthracene, 1-methyl-	52
70.875	0.7	Phenanthrene, 2-methyl-	83
		Phenanthrene, 1-methyl-	78
		Anthracene, 2-methyl-	78
72.472	3.5	Naphthalene, 2-phenyl-	95
75.947	0.59	1,2,4,8-Tetramethylbicyclo[6.3.0]undeca-2,4-diene	60
		Tricyclo[8.2.2.2(4,7)]hexadeca-2,4,6,8,10,12,13,15-octaene	35
		6-Phenylbenzocyclohepten-7-one	27
76.69	0.36	Tricyclo[8.2.2.2(4,7)]hexadeca-2,4	52
		,6,8,10,12,13,15-octaene	
		Naphthalene, 2-phenyl-	38
76.712	0.41	Tricyclo[8.2.2.2(4,7)]hexadeca-2,4	47
		,6,8,10,12,13,15-octaene	

		[1,1'-Biphenyl]-4,4'-dicarbonitril	44
		[1,1'-Biphenyl]-2,2'-dicarbonitril	38
78.346	2.26	Pyrene	94
		Fluoranthene	94
79.831	2.04	Fluoranthene	93
		Benzene, 1,1'-(1,3-butadiyne-1,4-diyl)bis-	70
81.12	0.56	Fluoranthene	46
		4,4'-Bis(tetrahydrothiopyran)	42
		Benzene, 1,1'-(1,3-butadiyne-1,4-diyl)bis-	41
81.154	0.16	Fluoranthene	46
		Pyrene	41
81.615	3.19	Pyrene	89
		Fluoranthene	89
88.385	0.06	11H-Benzo[b]fluorene	87
88.408	0.03	11H-Benzo[b]fluorene	55
		Fluoranthene, 2-methyl-	55

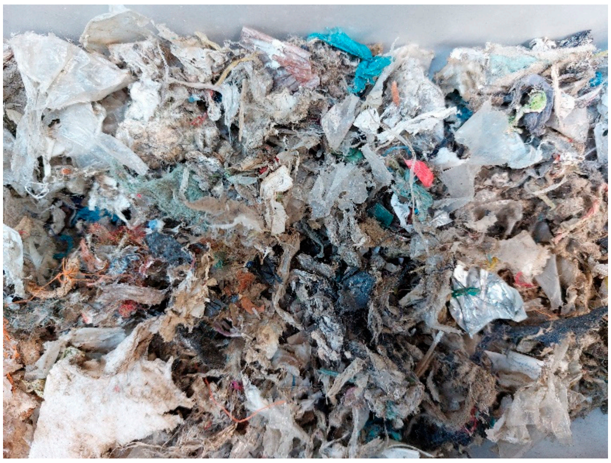

Fig. S2 RDF used as feedstock

Table S10-Proximate analysis results	
**RDF-raw**	
Humidity (wt.%)	36.03
Volatile (wt.%))	50.70
Volatile (wt.%) dry basis	79.24
Volatile (wt.%) dry ash free basis	89.29
Fixed Carbon (wt.%)	6.07
Fixed carbon (wt.%) dry basis	9.49
Fixed carbon (wt.%) dry ash free basis	10.69
Ashes (wt.%)	7.20
**RDF-dry**	
Humidity (wt.%)	3.74
Volatile (wt.%)	72.8
Volatile (wt.%) dry basis	75.63
Volatile (wt.%) dry ash free basis	89.06
Ashes (wt.%)	14.5
Fixed carbon (wt.%)	8.95
Fixed carbon (wt.%) dry basis	9.30
Fixed carbon (wt.%) dry ash free basis	10.94
**RDF-milled**	
Humidity (wt.%))	1.61
Volatile (wt.%)	80.7
Volatile (wt.%) dry basis	82.03
Volatile (wt.%) dry ash free basis	90.35
Ashes (wt.%)	9.05
Fixed carbon (wt.%)	8.70
Fixed carbon (wt.%) dry basis	8.84
Fixed carbon (wt.%) dry ash free basis	9.74

Table S11 Micro GC Column Technical Specifications (T = 70 °C).

Column	Type	Lenght (m)	Thickness (μm)	Carrier gas	Molecules detected
A	OV-1	8	1.2	He	C2-C6
B	Allumina	14	5	He	C2-C6
C	PLOT-U	8	3	He	C2H6, C2H4, C3H8, C3H6, CO2
D	MS5A	10	12	Ar	H2, CO, N2, CH4, O2

The gas used to carry the gaseous species through A, B, C columns is helium, while argon was used as carrier in column D. The column OV-1 and Alumina allow the determination of C_2-C_6 hydrocarbons. PLOT-U column gives the CH_4, C_2H_4, C_2H_6 and C_3H_6 concentration, while H_2, CO_2 and CO are determined through MS5A column.

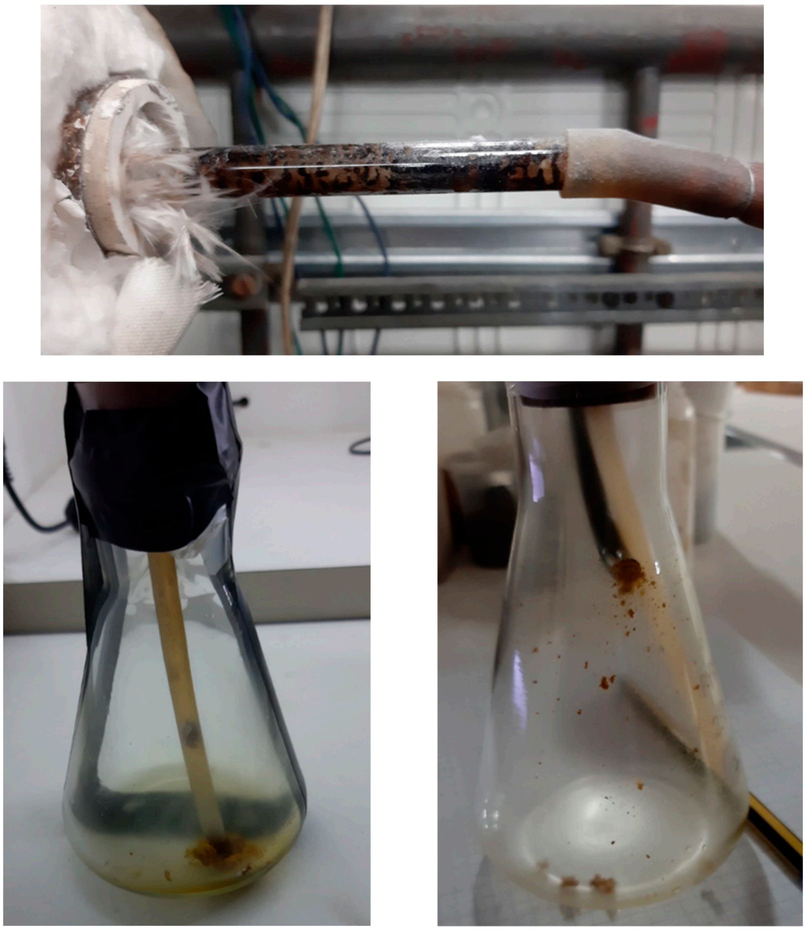

Figure S3: Waxy residue stacked on the reactor wall (up); Condensed residues collected in the flasks (down).
